# Timed fetal inflammation and postnatal hypoxia cause cortical white matter injury, interneuron imbalances, and behavioral deficits in a double-hit rat model of encephalopathy of prematurity^☆^

**DOI:** 10.1016/j.bbih.2024.100817

**Published:** 2024-07-05

**Authors:** M.J.V. Brandt, C.M. Kosmeijer, E.J.M. Achterberg, C.G.M. de Theije, C.H. Nijboer

**Affiliations:** a Department for Developmental Origins of Disease, University Medical Center Utrecht Brain Center and Wilhelmina Children's Hospital, Utrecht University, Lundlaan 6, 3584 EA, Utrecht, the Netherlands; b Department of Animals in Science and Society, Division of Behavioural Neuroscience, Faculty of Veterinary Medicine, Utrecht University, Yalelaan 2, 3584 CM, Utrecht, the Netherlands

**Keywords:** Encephalopathy of prematurity, Myelin deficits, Oligodendrocyte, Inflammation, Hypoxia

## Abstract

Extreme preterm birth-associated adversities are a major risk factor for aberrant brain development, known as encephalopathy of prematurity (EoP), which can lead to long-term neurodevelopmental impairments. Although progress in clinical care for preterm infants has markedly improved perinatal outcomes, there are currently no curative treatment options available to combat EoP. EoP has a multifactorial etiology, including but not limited to pre- or postnatal immune activation and oxygen fluctuations. Elucidating the underlying mechanisms of EoP and determining the efficacy of potential therapies relies on valid, clinically translatable experimental models that reflect the neurodevelopmental and pathophysiological hallmarks of EoP. Here, we expand on our double-hit rat model that can be used to study EoP disease mechanisms and therapeutic options in a preclinical setting. Pregnant Wistar dams were intraperitoneally injected with 10 μg/kg LPS on embryonic day (E)20 and offspring was subjected to hypoxia (140 min, 8% O_2_) at postnatal day 4. Rats exposed to fetal inflammation and postnatal hypoxia (FIPH) showed neurodevelopmental impairments, such as reduced nest-seeking ability, ultrasonic vocalizations, social engagement, and working memory, and increased anxiety and sensitivity. Impairments in myelination, oligodendrocyte maturation and interneuron development were examined as hallmarks for EoP, in different layers and coordinates of the cortex using histological and molecular techniques. Myelin density and complexity was decreased in the cortex, which partially coincided with a decrease in mature oligodendrocytes. Furthermore, interneuron populations (GAD67+ and PVALB+) were affected. To determine if the timing of inducing fetal inflammation affected the severity of EoP hallmarks in the cortex, multiple timepoints of fetal inflammation were compared. Inflammation at E20 combined with postnatal hypoxia gave the most severe EoP phenotype in the cortex. In conclusion, we present a double-hit rat model which displays various behavioral, anatomical and molecular hallmarks of EoP, including diffuse white matter injury. This double-hit model can be used to investigate pathophysiological mechanisms and potential therapies for EoP.

## Highlights

• This article explores a double-hit rat model for encephalopathy of prematurity.
• Fetal inflammation and postnatal hypoxia caused behavioral impairments in rats.
• Myelin expression, oligodendrocytes, interneurons and microglia were affected in the FIPH model.
• The timing of fetal inflammation determined the severity of anatomical hallmarks.

## Graphical abstract

**Figure:**
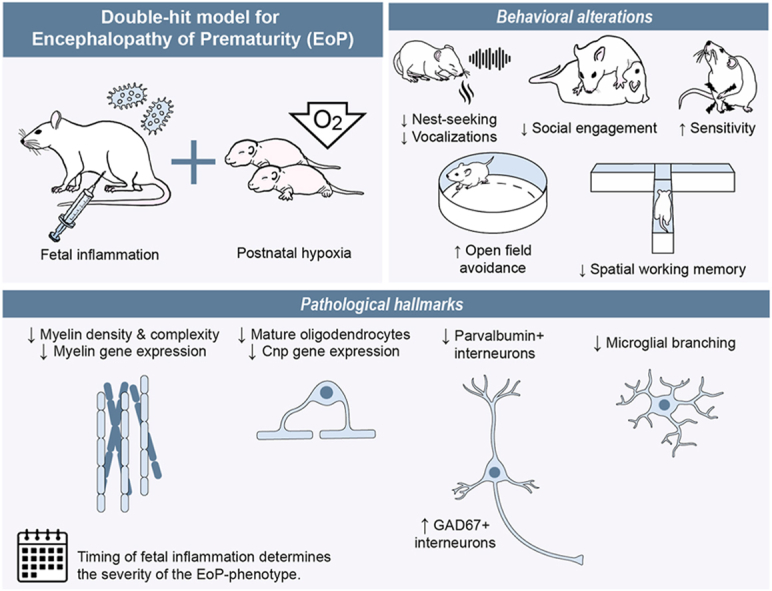

## 1 Introduction

With over 10% of infants born prematurely (before 37 weeks of gestational age) worldwide, brain injury resulting from preterm birth-associated adversities in early life is a significant problem in perinatal medicine. Especially after *extremely* preterm birth (< 28 weeks of gestation), long-term neurodevelopmental problems can arise, including sensory or motor impairments, social/emotional disorders, cognitive dysfunction, and problems in learning, memory, and/or attention (Chawanpaiboon et al., 2019; Vogel et al., 2018). Frequently, deficits in prematurely born infants span multiple neurodevelopmental domains (Back, 2014; Fleiss et al., 2020). Brain abnormalities acquired by the wide range of early-life adversities associated with preterm birth are collectively called encephalopathy of prematurity (EoP). The etiology of EoP is multifactorial with early termination of the intrauterine environment, antenatal or postnatal infections, cerebral oxygen disbalances, and suboptimal nutrition as major risk factors (Galinsky et al., 2018; Gilles et al., 2018). During the third trimester of pregnancy, the fetal brain progresses through critical developmental stages*,* making it especially vulnerable to preterm birth-associated insults (Hagberg et al., 2015). The specific timing and nature of these insults differs per patient and determines the degree of injury and/or maldevelopment of the brain (Ophelders et al., 2020), leading to a heterogeneous neuropathophysiology. In general, EoP encompasses both white and gray matter abnormalities, with gliosis, dysmaturation of oligodendrocytes and (inter)neurons, neuronal and axonal damage, and hypomyelination as the main pathophysiological hallmarks (Kinney and Volpe, 2018; Ophelders et al., 2020). Several brain areas are affected, including the white matter tracts, cerebral cortex, hippocampus, thalamus, basal ganglia, and cerebellum (Back, 2014; Back and Miller, 2014; Volpe, 2019). These brain morphological alterations often persist into adulthood, causing long-term functional morbidity (Johnson et al., 2010; Lean et al., 2017; Meng et al., 2016; Nosarti et al., 2008). Advances in neonatal care have markedly improved perinatal and neurological outcomes for preterm infants, however to date there are no curative therapeutic options available that directly target EoP disease mechanisms in the brain (Ramachandran and Nair, 2018; Vaes et al., 2021b). The validation of curative treatments for EoP relies on the optimization and characterization of clinically relevant experimental models that encompass the multifactorial etiology and multifaceted pathophysiology of EoP (Jantzie and Robinson, 2015; Kinney and Volpe, 2012; Ophelders et al., 2020).

Over the years, several animal models have been put forward to study EoP, that include either single or repeated inflammatory (e.g. Rousset et al., 2013; Schang et al., 2014; Drommelschmidt et al., 2017; Gussenhoven et al., 2018), hypoxic, or hyperoxic hits (e.g. Sifringer et al., 2010; Raymond et al., 2011). Despite the multifactorial etiology of EoP, few models incorporate a combination of multiple hits (e.g. Brehmer et al., 2012; Van Tilborg et al., 2018; Vaes et al., 2020). Indeed, it was shown that the risk for EoP increases with cumulative exposure to preterm-birth associated adversities, such as the development of necrotizing enterocolitis and an increased number of days requiring mechanical ventilation (Barnett et al., 2017). Additionally, to this day there are very few multiple-hit models available that mimic subtle and diffuse patterning of EoP that is more commonly seen in current patient cohorts (Ophelders et al., 2020). In this study, we investigated the functional and pathophysiological hallmarks of EoP in a rat model that combines two clinically relevant hits during appropriate stages of brain development, i.e. lipopolysaccharide (LPS)-induced fetal inflammation (FI) at embryonic day (E)20 and postnatal hypoxia (PH) at postnatal day (P)4 (i.e. FIPH model). These timepoints were chosen to represent the human preterm brain during the second and early third trimester of human pregnancy due to the homologous developmental processes that occur in rats and humans at this stage (Markova et al., 2019; Workman et al., 2013; Zeiss, 2021). Our model paradigm reflects major anatomical, molecular, and behavioral hallmarks of EoP, including hypomyelination, impaired oligodendrocyte maturation, reduced parvalbumin (PVALB)-positive interneuron density, alterations in total interneuron density and microglial morphology, and behavioral changes persisting until adulthood. Moreover, we systematically investigated the effect of *timing* of the fetal inflammatory insult on these pathophysiological hallmarks of EoP across layers I-IV and V-VI of the rostral and caudal rat cortex by inducing fetal inflammation at E15+16 (Fortier et al., 2007; Cui et al., 2009), E18+19 (Van Tilborg et al., 2018) or E20. As hypothesized, differences in pathophysiological hallmarks are dependent on the gestational timing of inflammation, which gives insight into the heterogeneous pathophysiology of EoP. In future, the E20 FIPH model can be used to study disease mechanisms and candidate therapies to optimize neurodevelopmental outcome for babies born preterm.

## 2 Materials & methods

### 2.1 Ethical considerations

This study was conducted in accordance with institutional guidelines for the care and use of laboratory animals of Utrecht University and the University Medical Center Utrecht, and all animal procedures related to the purpose of the research were approved by the local Animal Welfare Body (AWB; Utrecht, the Netherlands) under an ethical license provided by the national competent authority (*Centrale Commissie Dierproeven*, CCD, the Netherlands), securing full compliance with the European Directive 2010/63/EU for the use of animals for scientific purposes. The experimental design was subsequently approved by the AWB. All efforts were made to report animal experiments according to the ARRIVE guidelines (Percie du Sert et al., 2020).

### 2.2 Animals

Adult Wistar female and male rats (RccHan:WIST) were ordered (Envigo, the Netherlands) and arrived at the animal facility at around 10 weeks old. Animals were housed in groups of 2–3 in open cages (Eurostandard Type IV S, 210 x 375 × 480 mm [height x width x depth, H x W x D]) with woodchip bedding, a plastic shelter, wooden chewing blocks, and paper tissues provided, on a 12h day/night cycle (lights on at 7:00 a.m.), in a temperature-controlled room at 20–24°C and 45–65% humidity. Water and food (Rat/mouse maintenance, V1535-000, Ssniff Spezialdiäten GMBH, Germany), were available *ad libitum*. After at least one week of habituation, female rats’ estrous cycles were determined using a vaginal impedance monitor (59160, Stoelting Europe, Ireland). If estrous was established (value higher than 4.0 kΩ or highest relative value out of 4 days), females were time-paired with a male in a 1:1 ratio for 24 h, and this day was recorded as embryonic day (E) 0. After time-pairing, rats were housed in same-sex pairs. Female and male rats were used for breeding until they reached 9 months of age.

### 2.3 Double-hit rat FIPH model

Fetal inflammation was induced by intraperitoneally (i.p.) injecting timed-pregnant dams with 3–10 μg/kg lipopolysaccharide (LPS; UltraPure, from *Escherichia coli* 055:B5, lot#: 9588-42-01, #tlrl-b5lps, InvivoGen, USA) in 1 ml/kg 0.9% sodium chloride (NaCl) at E15 and 16 (3 μg/kg; Fortier et al., 2007; Cui et al., 2009), E18 and 19 (3 μg/kg; van Tilborg et al., 2018), or E20 (10 μg/kg). Only female rats naïve to LPS were used, to prevent potential endotoxin tolerance (Liu et al., 2019). The optimal LPS dosage for at least 75% litter survival with this new LPS was established by first titrating LPS dosages (Supplementary Fig. 1), as the lot number of LPS used in previous studies (from *E.Coli* O55:B5, L2880, Sigma; van Tilborg et al., 2018; Vaes et al., 2020) was no longer commercially available, and it has previously been shown that the dosage of immune-activating stimuli during pregnancy may have different effects across different manufacturers and production batches (e.g. Kentner et al., 2019). UltraPure LPS was used to reduce batch-to-batch variation and improve reproducibility in future (Hirschfeld et al., 2000; Parusel et al., 2017). Control animals were injected with 1 ml/kg 0.9% NaCl on the same embryonic days as LPS-injections for each model. All injections were performed between 9:00–11:00 a.m. Dams were randomly allocated to the LPS or NaCl injection group using an online randomization tool. After the injections, females were housed individually to give birth, and additional wood shavings and tissues were provided as nesting material.

The day of birth at E22 or E23 was considered as P0. At P1, pups were counted, and litters were randomly culled to a maximum of 8–10 pups (if possible 50/50 ratio male/female) to ensure adequate feeding of each pup. At P4, pups were placed in the hypoxia setup, consisting of a temperature-controlled chamber (ThermaCage MK3, BioServices, the Netherlands) equipped with a feedback-controlled heat mat, that was used to keep an inner chamber at a constant sensor temperature of 36.0–36.5 °C (approximate core temperature between 37.7 and 38.2 °C as observed in pilot experiments, data not shown). A thermometer controlling the heat mat was placed at the bottom of the chamber and made inaccessible to pups to ensure stable measurements. Pups were exposed to hypoxia for 140 min by exposure to humidified 8% O_2_/92% N_2_ into the airtight inner chamber (Van Tilborg et al., 2018). After a brief recovery period (∼5 min), pups were placed back into the home cage. The survival rate of rat pups after hypoxia was >98%. Control pups were not subjected to hypoxia at P4.

Prior to the start of the study, appropriate sample sizes were calculated separately for behavioral, molecular and histological analysis based on estimated effects sizes and/or previous experience. However, group size and composition were in practice partially determined by the number of litters, amount of pups, and sex distribution within litters. The total number of animals used, as well as the allocation of males and females in each group for each of the analyses can be found in Supplementary Table 1.

### 2.4 Behavioral assessments

For long-term behavioral assessments, rats were weaned around P26, separating males and females but keeping same-sex littermates together housed in groups of two or three. All behavioral tests and analyses were performed by experienced researchers that were blinded to the experimental conditions. Each test was consistently performed within a specific time window during the day, and the order of testing was counterbalanced between groups and sexes, to ensure the time of day would not influence the results. In between trials, all testing equipment was cleaned with soapy water and/or ethanol, and thoroughly dried to eliminate olfactory cues. Except for the direct social interaction test, which was performed under red light conditions (normal day/night cycle), all behavioral tasks were performed under normal light conditions. Rat were habituated to the experimental chamber and to handling prior to behavioral testing, to familiarize the animals with the procedures and to reduce stress.

#### 2.4.1 Nest-seeking task

At P8, pups were placed in the middle of a plexiglass arena (19 x 21 × 37 cm [H x W x D]) that was divided into equal thirds lengthwise. Clean, unused bedding material was placed on one end and nest bedding from the home cage containing three maternal fecal boli was placed on the opposite end. The pup was placed in the center of the arena, facing neither type of bedding. The time it took a pup to cross the line to the side containing the home cage bedding with both the head and the forepaws was recorded, with a maximum trial duration of 60 s. After 60 s, the pup was placed in a separate cage during which the arena was reversely oriented. After a latency of 30 s, the trial was repeated for each pup. The average latency to find the nest over two trials was reported.

#### 2.4.2 Ultrasonic vocalizations

On P8, P10 and P12, pups were individually placed in the center of a soundproofed cabinet (EKET, IKEA, Sweden) and vocalizations were recorded using an ultrasonic microphone (M500-384, Pettersson, Sweden) and Audacity software (V3.2, Audacity Team, 2021) for a total duration of 5 min, after which the pup was immediately placed back in the home cage. The temperature was kept constant at RT and was equal to standard home cage conditions. Audio was converted into wav format and imported into DeepSqueak (Coffey et al., 2019) for MatLab (MathWorks, USA). Vocalizations were detected in DeepSqueak using the “Rat_detector_YOLO_R1” classifier, detecting all calls between 20 and 120 kHz with a score threshold of 0 for a total duration of 300 s. Next, all calls from a randomly chosen subset of animals was manually classified into different call categories (short, chevron, upward, downward, flat, complex) by an experienced observer. Using this subset, a classifier model was trained to recognize different call classes with an accuracy of 79%. Finally, all audio fragments were automatically classified using the trained neural network.

#### 2.4.3 Direct social interaction

Social play behavior was assessed in rats as previously described (Achterberg et al., 2014; Trezza et al., 2011; Van Tilborg et al., 2018), between the ages of P34–P37. On the two days before the trial, rats from the same home cage were habituated to a custom-made plexiglass play arena (60 x 40 × 40 cm [H x W x D]) filled with woodchip bedding and left undisturbed for 10 min under red light conditions. Males and females were habituated and tested in separate arenas. On the trial day, rats were socially isolated under normal light conditions for 2.5 h before testing in separate cages containing woodchip bedding, tissues, a plastic shelter, a chewing block, and food and water *ad libitum*. After social isolation, paired unfamiliar rats were simultaneously placed in the play arena, and left to play undisturbed for 15 min under red light conditions. Rat pairs were made between unfamiliar animals of the same experimental group (FIPH or control), of the same sex, with an average body weight difference of <5% and an age difference of 0–3 days. Behavior was assessed per couple, by scoring the frequency and duration of 1) typical play behaviors: pinning, pouncing, boxing, and chasing, 2) general social exploration, and 3) non-social (cage) exploration using The Observer XT software (XT16, Noldus, the Netherlands). Social engagement was calculated as the total amount of time spent on all social behaviors combined (pinning, pouncing, boxing, following, social exploration).

#### 2.4.4 Open field test

The open field test was used to investigate locomotor activity and anxiety-like behavior in rats at P39-40 (Gould et al., 2009). Animals were individually placed in the middle of an opaque, circular arena (Ø100 cm, height: 34 cm) and allowed to explore undisturbed for 15 min. The rat was automatically tracked using Ethovision XT software (version 9.0.718; Noldus, the Netherlands). For analysis, the arena was divided in an inner (Ø 75 cm) and an outer zone (12.5 cm from the wall). The number of transitions from the outer to the inner zone and the time spent in the inner and outer zones were quantified. Additionally, total distance moved was recorded as a measure for overall locomotor activity.

#### 2.4.5 Adhesive removal test

To assess potential sensory or motor impairments, the adhesive removal test was performed at P40-41. Rats were habituated to the testing room 15 min prior to the testing. Before each testing sequence, the rat was placed in the testing cylinder for a 60 s habituation period. A round adhesive sticker (10 mm diameter, ToughSpots™, DiversifiedBiotech, USA) was placed with equal pressure on one forepaw of the animal, thereby covering the interdigital pads, thenar and hypothenar of the ventral hairless plane of the forepaw. The animal was then placed in a round plexiglass transparent colorless cylinder (Ø 20 cm, H: 30 cm) and the time to contact the sticker, determined as a paw shake or nose-to-sticker contact, and the time to remove the adhesive sticker was measured, with a maximum trial duration of 120 s. This was repeated for the other forepaw. Each forepaw was tested alternately for a total of four stickers per paw, data is presented as the average of eight trials.

#### 2.4.6 Spontaneous alternation T-maze

Short-term spatial working memory was assessed between 6 and 7 weeks of age by performing the spontaneous alternation T-maze test (Deacon and Rawlins, 2006; d’Isa et al., 2021). The T-shaped apparatus was made of opaque acrylic panels, with each arm measuring 40 x 10 × 50 cm (H x W x D) connected in a T-shape leaving a center zone of 10 cm^2^, with sliding panels to close the two opposing goal arms. One trial consisted of a sample run and a choice run. For the sample run, the animal was placed in the start arm and was free to enter either of the goal arms with a maximum trial duration of 120 s. When the animal entered either goal arm with all four legs, the animal was confined to that arm by closing the division panel and was allowed to explore the arm for 30 s. After this sample run, the animal was placed in a separate cage for exactly 30 s, allowing the researcher to open all arms again. After this interval, the animal was again placed in the start arm for the choice run, and a positive score for spontaneous alternation was awarded if the animal chose the opposite arm from the one explored in the sample run. For each run the latency to entry of a goal arm was recorded. Each animal was tested for a total of 8 separate trials, one morning and one afternoon trial for four consecutive days. Preference for the alternating arm is expressed as the percentage of correct alternations over eight trials.

#### 2.4.7 Tapered beam walk

To assess motor coordination and balance, rats between 7 and 8 weeks of age were trained and tested on an elevated tapered beam (length = 175 cm, width ranging from 6 cm at the start to 1.5 cm at the end). At 2 cm below the beam's top surface, a safety ledge protruding for 2 cm on either side of the beam was placed on the first training day and the two testing days. During the second training day this support ledge was removed to prevent animals of using the support ledge when it was not necessary. Each trial consisted of four subsequent runs per animal each day. Each trial started with a 2-min habituation period where the animal was placed in a dark box connected to the narrow end of the beam, containing woodchip bedding, a plastic shelter, and two paper tissues. To start a run, the animal was placed on the wide end of the beam. After each run, the animal was left in the dark box for 1 min. Over the two training days, the rats were trained to run over the full length of the beam without stopping. For the two testing days, rats performed one trial daily of which the first of the four runs was not recorded. All measurements were taken from the middle 120 cm of the beam, omitting the first 30 cm and last 25 cm of the beam, to give an accurate representation of continuous movement. Recordings were manually scored for the number of steps per foot, and the number of full-slips (score of 1; foot completely off the beam and/or on the safety ledge) and half-slips (score of 0.5; foot grabbing the side of the beam with 3 or more toes) per foot. The average percentage of foot faults per step over six trials was calculated. Since we did not observe differences in the direction of the data when separating between right or left limbs, or hind or front limbs, the average foot faults per step of all four limbs was analyzed. Additionally, the traverse time was measured.

### 2.5 Immunohistochemistry

Animals were euthanized at P20 by injecting 20% pentobarbital i.p., and transcardially perfused with phosphate buffered saline (PBS) followed by 4% paraformaldehyde (PFA). Perfused brains were harvested and fixated in 4% PFA overnight, dehydrated, embedded in paraffin and cut into 8 μm coronal sections at rostral (*bregma* +0.48 mm) and caudal (*bregma* −3.3 mm) coordinates. After rehydration, sections were heated to 95 °C in sodium citrate buffer (10 mM, pH 6.0) for antigen retrieval, blocked with bovine serum albumin or normal goat serum, and incubated overnight with primary antibodies for Myelin Basic Protein (MBP; 1:2000, ab218011, Abcam, UK) to stain myelin fibers, Oligodendrocyte Transcription Factor 2 (OLIG2; 1:500, AB9610, Chemicon, MilliporeSigma, USA) to stain for oligodendrocyte-lineage cells, Quaking 7 binding antibody CC1 (CC1; 1:300, OP80, Calbiochem, MilliporeSigma, USA) to stain for myelinating oligodendrocytes, ionized calcium binding adapter molecule 1 (IBA1; 1:2000, 019–19741, FUJIFILM Wako Chemicals, USA) to stain microglia, parvalbumin (PVALB; 1:1000, P3088, MilliporeSigma, USA) to stain for PVALB+ interneurons, glutamate carboxylase 67 (GAD67; 1:500, mab5406, MilliporeSigma, USA) to stain for total interneurons, and COUP-TF interacting protein 2 (CTIP2; 1:1000, ab18465, Abcam, UK) to distinguish neuronal layers V-VI. The next day, slides were incubated with the appropriate secondary antibodies (1:500, goat-anti-rabbit Alexa Fluor [AF] 594 [MBP], goat-anti-mouse AF488 [CC1, GAD67, PV], goat-anti-rat AF488 [CTIP2], goat-anti-rat AF647 [CTIP2], or goat-anti-rabbit AF568 [IBA1, OLIG2]; A11012, A11001, A11006, A21247, Invitrogen, ThermoFisher Scientific, USA and ab175471, Abcam, UK), counterstained with 4′,6-dia-midino-2-fenylindool (DAPI; 1:5000, D9542, MilliporeSigma, USA) for nuclear staining, and embedded in FluorSave (345789, MilliporeSigma, USA).

Fluorescence images were obtained at 2.5× magnification in both hemispheres of the cortex for cortical myelin (Fig. 2H). As no differences were found between the two hemispheres in cortical myelin expression in either experimental group (data not shown), the fluorescent images at 10x (PVALB, OLIG2/CC1) and 20× magnification (MBP, IBA1), were obtained from one hemisphere in two locations of the primary somatosensory cortex (S1, *bregma* +0.48 mm: mediolateral cortex: forelimb area, lateral cortex: barrel field; *bregma* −3.3 mm: mediolateral cortex: trunk area, lateral cortex: barrel field) in the CTIP2-positive (deeper cortical layers V-VI) and CTIP2-negative layers (upper cortical layers I-IV), using an Axio Observer Z1 microscope with Zen Blue software (Carl Zeiss, Germany, for locations see Fig. 2M-N). Imaging and image analysis were performed by researchers who were blinded to the experimental conditions.

### 2.6 Image analysis

For the MBP analysis, 2.5× magnification images were analyzed in a semi-automatic manner using a custom macro in Fiji (ImageJ v1.53C, US National Institutes of Health, USA). Briefly, the area of whole cortex and CTIP2+ cortex (corresponding to layers V and VI) were manually drawn using the polygonal selection tool. A manual threshold was applied to the MBP+ signal, and the percentage of MBP+ area was calculated relative to the total area of CTIP2+ and CTIP2- layers. The percentage of MBP+ area was averaged between both hemispheres for each animal.

In the MBP microstructure analysis, a binary image was generated using Fiji software, by setting a manual threshold for 20x images (two per location) based on MBP+ signal. Binary images were preprocessed using the DiameterJ plugin (v1.018) segmentation feature, after which smoothened images were skeletonized and the number of intersections per image was calculated (Van Tilborg et al., 2017). Two images from the CTIP2+ and CTIP2- layers were averaged per brain region (mediolateral or lateral cortex area).

OLIG2+/CC1+ cell count and colocalization in 10x images (one per location) was analyzed using a custom CellProfiler pipeline to identify OLIG2+ cells (Carpenter et al., 2006), available upon request. Cytoplasmic CC1+ signal was measured in a standardized perimeter around OLIG2+ nuclei. Object intensity, size, shape, and texture, were used to train a classifier to a predicted accuracy of >90%, using CellProfiler Analyst software (version 4.2.1, Stirling et al., 2021). This classifier was utilized to quantify the total number of OLIG2+ cells and the number of CC1+/OLIG2+ cells, which was used to calculate the percentage of mature (CC1+/OLIG2+) oligodendrocytes (OLIG2+).

PVALB+ cells in 10x images (one per location) were counted with a custom CellProfiler pipeline which identified the number of DAPI+/PVALB+ double-positive nuclei. GAD67+ were manually quantified by an experienced observer who was blinded to the experimental condition.

Cell count and morphometric analysis of IBA1+ cells in 20x images (two per location) was performed using a custom pipeline in CellProfiler. In short, IBA1+/DAPI+ double-positive nuclei were identified and used as the center of IBA1+ cells from which cell processes were automatically traced, based on the signal threshold. Cell count, size and shape (Feret's diameter, perimeter) were measured before performing a skeleton analysis to obtain the number of branch endpoints for each IBA1+/DAPI+ cell.

### 2.7 Reverse transcription quantitative polymerase chain reaction (RT-qPCR)

Animals within each litter were randomly allocated towards termination at P8, P12, or P16. Pups were euthanized by i.p. injecting 20% pentobarbital. Whole cerebrums were immediately extracted and snap-frozen in liquid nitrogen. Tissues were kept at −80 °C until further analysis.

Following the protocol of Wager-miller et al. (2020), 1 mm coronal sections were made from the frozen rat cerebrums using brain matrices (RBM-3000C and RBM-4000C, ASI instruments, USA), and arranged rostral to caudal on a frozen glass plate. The somatosensory cortex of the obtained coronal sections around *bregma* −3.3 mm was dissected using a cold scalpel (Fig. 2B) and stored in Eppendorf tubes at −80 °C until further processing. For P12 and P16, the somatosensory cortex around −3.3 *bregma* was isolated from two 1 mm sections, and at P8 from one 1 mm section. Around *bregma* +0.48 mm, the somatosensory cortex was isolated using precision brain punches (Ø 1.25-1.25-1.5 mm for P8-12-16 brains respectively, Supplementary Fig. 2A; Ted Pella, Inc., CA, USA). In all cases, the tissue was collected and pooled from both hemispheres.

Total RNA was isolated from the cortical tissue using the RNeasy Mini Kit (74104, Qiagen, the Netherlands), and cDNA was synthesized with iScript™ Reverse Transcription Supermix for RT-qPCR (1708840, Bio-Rad, USA). RT-qPCR was carried out with SYBR™ Select Master Mix (4472908, ThermoFisher Scientific, USA) and the QuantStudio™ 3 Real-Time PCR System (A28136, ThermoFisher Scientific, USA) using primers for hypoxanthine phosphoribosyltransferase 1 (*Hprt1*), phosphoglycerate kinase 1 (*Pgk1*), myelin-associated glycoprotein (*Mag*), myelin basic protein (*Mbp*), myelin-associated oligodendrocyte basic protein (*Mobp*), myelin oligodendrocyte glycoprotein (*Mog*), proteolipid protein 1 (Plp1), platelet derived growth factor alpha (*Pdgfra*), oligodendrocyte transcription factor 2 (*Olig2*), 2′,3′-cyclic nucleotide 3′ phophodiesterase (*Cnp*), parvalbumin (*Pvalb*), glutamate decarboxylase 67 (*Gad67*; all primer sequences can be found in Supplementary Table 2). Gene expression levels were normalized to the mean Ct of reference genes *Hprt1* and *Pgk1* (stable and equivalent expression of reference genes between both groups on all timepoints was confirmed with an average Ct of 20.723/20.850 control/FIPH and 22.554/22.665 control/FIPH, respectively), and to the mean of the control group at the earliest timepoint to obtain the ΔΔCt. Fold change was calculated as 2^−ΔΔCt^.

### 2.8 Statistical analysis

Data was analyzed using IBM SPSS Statistics (v.27.0, IBM, USA). Prior to formal analysis, data was tested for potential outliers using the ROUT method (Q = 1%), and outliers who met this criterion were omitted. After outlier removal, data was tested for normality and homogeneity of variance using the Shapiro-Wilk test and Levene's test, respectively. For qPCR data, ΔΔCt values were statistically analyzed. Fold change-values were plotted in graphs for ease of interpretation.

Behavioral data were analyzed using an unpaired *t*-test (or nonparametric equivalent) or a mixed model (experimental group × test day). Histological data were analyzed using a robust two-way (experimental group × brain area) mixed model separately for each FIPH model (E15+16, E18+19 or E20). These analyses were followed by planned multiple comparisons between the control group and FIPH groups per brain coordinate (*bregma* +0.48 mm or −3.3 mm) and cortical layers (I-IV or V-VI) per cortical area using Šidák's method. Gene expression data were analyzed using a robust two-way general linear model (experimental group × timepoint), followed by pairwise comparisons between experimental groups within each. Data was reported as statistically significant when p < 0.05. P-values of pairwise comparisons are reported in the text, other test statistics can be found in Supplementary Tables 3–6.

For multivariate analysis of variance (MANOVA) and discriminant function analysis (DFA), histological data from different experimental cohorts were normalized to their respective control groups. Based on predefined hypotheses, the number of MBP+ intersections, the number of OLIG2+ cells, the percentage of CC1+/OLIG2+ cells, the number of PVALB+ cells and the number of IBA1+ endpoints per cell were chosen to represent the pathophysiological hallmarks of EoP. Images taken in the middle of the examined cortical regions (in the primary somatosensory cortex, see Fig. 6F) were chosen to represent the cortex. Separate MANOVAs and DFAs were run per combination of brain coordinate (*bregma* +0.48 mm or −3.3 mm) and cortical layer (I-IV and V-VI; a total of four separate tests). Mahalanobis distances were calculated for the combination of these five variables in these areas, and multivariate outliers with a Mahalanobis distance corresponding to a significance value of p < 0.001 were omitted. Before running the MANOVA, assumptions were checked for each of these variables using the Kolmogorov-Smirnov test for normality and Levene's test for equality of variances. Equality of covariance matrices was examined using Box's test, and covariances were accepted as equal when p > 0.001. Data was deemed statistically significant when Pillai's Trace <0.05 or Wilk's Lambda <0.05 for the MANOVA and DFA, respectively. Multivariate analyses were followed up by pairwise comparisons between FIPH animals and respective controls per model paradigm using using Šidák's correction method.

Graphs were created using Graphpad Prism software (v.9.3, USA) and shown as mean ± standard error of the mean (SEM).

## 3 Results

### 3.1 Fetal inflammation at E20 and postnatal hypoxia at P4 causes short- and long-term functional deficits

**Fig. 1:**
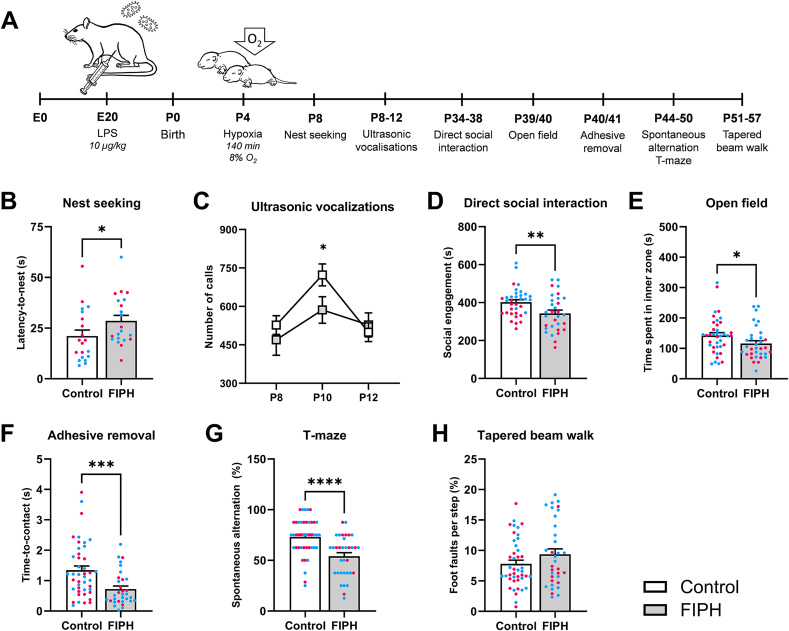
**Fetal inflammation at E20 and postnatal hypoxia at P4 causes neurodevelopmental and behavioral impairments**. A) Timeline of FIPH model induction and the behavioral test battery. B) FIPH animals took longer to locate the home cage bedding during the nest seeking task at P8. C) FIPH animals made less vocalizations during maternal separation at P10. D) During the direct social interaction test, FIPH couples spent less time on social engagement compared to controls. E) FIPH rats spent less time in the inner zone of the arena during the open field test. F) In the adhesive removal test, FIPH animals had a lower time-to-contact than controls, indicating increased sensitivity. G) In the spontaneous alternation T-maze, FIPH animals showed less preference for the alternating arm, compared to controls. H) No significant difference in foot faults on the tapered beam walk was found between FIPH and control animals. Data is presented as mean ± SEM. For the individual data points, magenta circles represent female and blue circles male rats. * p < .05, ** p < .01, *** p < .001, **** p <.0001. E, embryonic day; FIPH, fetal inflammation and postnatal hypoxia; P, postnatal day; s, seconds. Created with BioRender.com.

To determine whether FIPH rats displayed functional deficits, a series of behavioral tests was performed across the developmental, social, sensorimotor and cognitive domains (Fig. 1A).

**Fig. 2:**
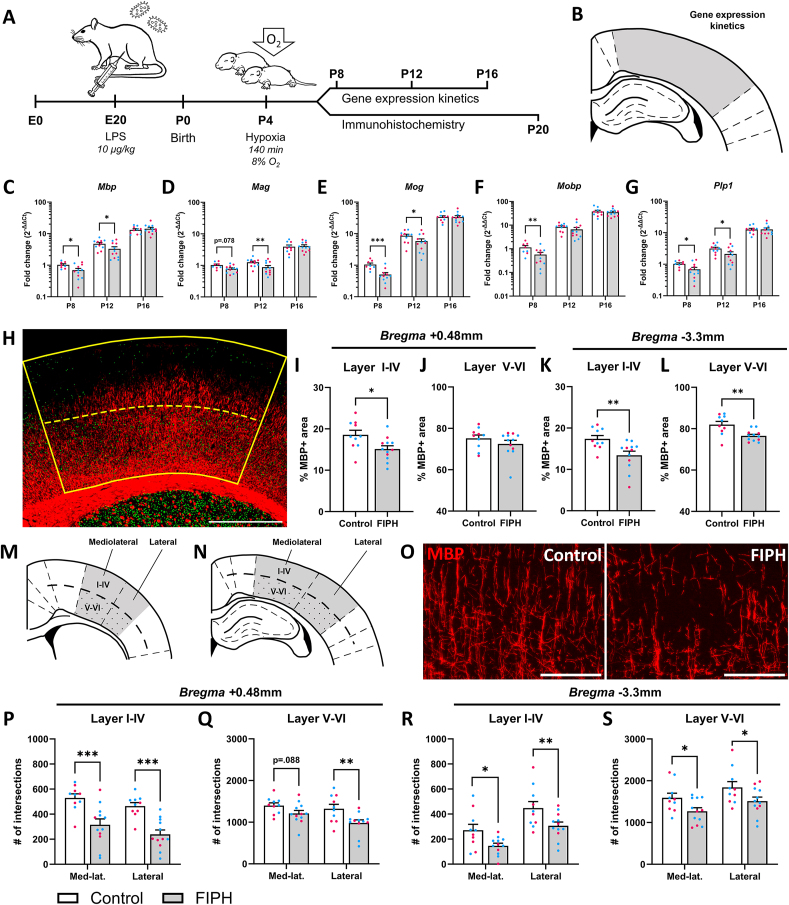
**Myelin expression is significantly impaired in FIPH animals compared to controls**. Fetal inflammation was induced on E20 and pups were subjected to postnatal hypoxia at P4. Brains were taken out for gene expression kinetic analysis on P8, P12 or P16 or for immunohistochemistry on P20 (A). Gene expression of myelin-related genes in the cortex at bregma -3.3mm (B) was reduced between P8 and P12 in FIPH animals compared to controls (C-G). Global myelin expression (red) measured in CTIP2+ (green) and CTIP2- areas (dashed line, H) was decreased in layers I-IV of the cortex of FIPH animals at bregma +0.48 (I), but not in layers V-VI (J), and in layers I-IV and V-VI at bregma -3.3mm (K-L). Myelin microstructure was imaged in layers I-IV or V-VI (dotted) of the mediolateral and lateral cortex at bregma +0.48mm (M) and -3.3mm (N). (O) Representative images of control (left) and FIPH (right) myelin microstructure in the mediolateral cortex. Myelin microstructure was affected in the mediolateral and/or lateral cortex at both bregma +0.48 (P, Q) and -3.3mm (R, S) in all layers. Data is presented as mean ± SEM. For the individual data points, magenta circles represent female and blue circles male rats. Scalebar figure H = 1000μm, scalebars figure O = 150μm, * p < .05, ** p < .01, *** p < .001. Gene expression data is plotted on a log10-scale. Figure A was created with BioRender.com. (For interpretation of the references to color in this figure legend, the reader is referred to the Web version of this article.)

In the early postnatal weeks, FIPH rat pups showed higher latency to find the home cage nest-bedding material at P8 compared to controls (p = 0.024, Fig. 1B). Additionally, FIPH pups made less ultrasonic vocalizations than control pups during a period of maternal separation at P10 (p = 0.033), but not at P8 or P12 (p = 0.371 and p = 0.656, respectively, Fig. 1C). The duration and frequency (kHz) of calls, or the type of calls, was not different between FIPH pups and controls (data not shown).

At (pre)pubertal age, non-familiar FIPH couples spent less time on social engagement than control couples (p = 0.007) in the direct social interaction task (Fig. 1D). No differences in the incidence of play behaviors (pinning and pouncing) or the time spent on social play were observed between FIPH and control couples (data not shown).

Locomotion and anxiety were assessed in the open field test, during which FIPH rats spent less time in the inner zone of the arena than controls (p = 0.031, Fig. 1E). No differences were found in the distance moved between FIPH and controls in the open field (data not shown), indicating that less time spent in the inner zone of the arena by FIPH rats was not due to decreased locomotion.

Sensorimotor function was tested with the adhesive removal test on the forepaws. Although FIPH rats removed the sticker within the same timeframe as controls (p = 0.366, data not shown), their time-to-contact was significantly shorter (p < 0.001, Fig. 1F), suggesting an increased sensitivity to sensory stimuli.

In the T-maze, a test for spatial working memory relying on the natural explorative behavior of rodents, controls showed a 74% preference for the unexplored arm while FIPH rats spontaneously alternated arms in only 54% of eight trials, which is around chance level (control vs. FIPH: p < 0.001, Fig. 1G). No differences were found between experimental groups on the latency to enter an arm during the sample run or during the choice run (data not shown).

Finally, in the tapered beam walk test for motor coordination and balance, there were no differences between FIPH and control animals on the mean number of foot faults (p = 0.258, Fig. 1H) or the traverse time (data not shown). No body weight differences were observed between groups during behavioral testing (data not shown). Combined, these data indicate that FIPH animals display developmental, social, sensory, and cognitive behavioral aberrations.

### 3.2 Developmental myelin deficits are induced by fetal inflammation and postnatal hypoxia

Since FIPH rats displayed behavioral deficits associated with EoP, we next examined potential myelin aberrations in the cerebral cortex. First, expression of genes involved in myelin production were assessed at P8, P12, and P16 in control and FIPH cortices around *bregma* −3.3 mm (Fig. 2A–B). *Mbp*, *Mog* and *Plp1* gene expression was decreased at P8 (p = 0.013, p < 0.001 and p = 0.013, respectively) and P12 (p = 0.011, p = 0.014 and p = 0.010, respectively), while *Mobp* and *Mag* gene expression was decreased on P8 or P12 only (p = 0.002 and p = 0.002, respectively) in FIPH pups compared to control pups, indicating early deficits in myelin production (Fig. 2C–G). Similarly, expression of all myelin-related genes was reduced at P12 at *bregma* +0.48 mm (Supplementary Fig. 2).

Since myelin-gene expression was reduced during development, we next investigated whether this resulted in anatomical cortical myelin deficits at P20. Two distinct bregma levels (i.e. *bregma* +0.48 and −3.3 mm) were chosen to examine the spatial dispersion of white matter aberrations in the cortex. Global investigation of cortical myelin density, using CTIP2+ as a marker to distinguish cortical layers V-VI (Fig. 2H), revealed that FIPH animals showed a reduced MBP+ density in layers I-IV at *bregma* +0.48 mm (p = 0.018), but not in layers V-VI (p = 0.273, Fig. 2I–J). At *bregma* −3.3 mm, global myelin expression was reduced in both layers I-IV (p = 0.007) and V-VI (p = 0.004, Fig. 2K-L) in FIPH rats compared to controls. Moreover, when examining myelin at a microstructural level (Fig. 2M-O), FIPH animals showed less complex myelin microstructure. The mediolateral and lateral cortex was affected across *bregma* +0.48 mm and −3.3 mm in all but one location (*bregma* +0.48 mm layers I-IV: p < 0.001 and p < 0.001, layers V-VI: p = 0.088 [n.s.] and p = 0.003, *bregma* −3.3 mm layers I-IV, p = 0.019 and p = 0.008, layers V-VI: p = 0.033 and p = 0.028, respectively, Fig. 2P–S). In sum, the data in Fig. 2 shows that combined fetal inflammation and postnatal hypoxia induced myelin deficits throughout the cortex.

### 3.3 Oligodendrocyte maturation was reduced after fetal inflammation and postnatal hypoxia

**Fig. 3:**
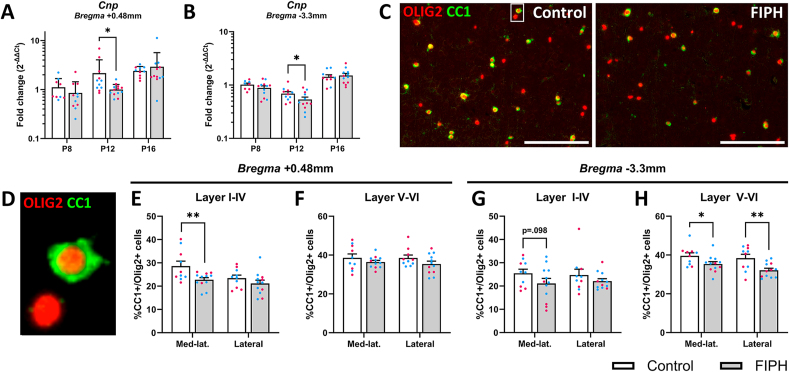
**Oligodendrocyte maturation is significantly impaired in FIPH pups compared to controls.** Fetal inflammation at E20 and postnatal hypoxia at P4 reduced cortical expression of the myelin production-associated enzyme Cnp at P12 in FIPH animals compared to controls at bregma +0.48mm (A) and -3.3mm (B). (C) Representative images of the number of mature (CC1+, green) oligodendrocyte-lineage (OLIG2+, red) cells in the mediolateral cortex of control and FIPH animals at P20. D) Magnification of an OLIG2+ and an OLIG2+/CC1+ cell (C, control). The number of mature (CC1+/OLIG2+) oligodendrocytes was reduced in layers I-IV (E) but not in layers V-VI (F) at bregma +0.48, and in layers V-VI at bregma -3.3mm (H) but not significantly in layers I-IV (G). Data is presented as mean ± SEM. For the individual data points, magenta circles represent female and blue circles male rats. Scalebar = 150μm, * p < .05, ** p < .01. Gene expression data is plotted on a log10-scale. (For interpretation of the references to color in this figure legend, the reader is referred to the Web version of this article.)

To investigate whether reductions in myelin complexity were related to an oligodendrocyte maturational deficit, gene expression of oligodendrocyte maturational stages was examined in the cortex during development. Expression of oligodendrocyte progenitor cell (OPC) marker *Pdgfra* and oligodendrocyte-lineage marker *Olig2* were not affected by FIPH on P8, P12, or P16 (all p > 0.05, Supplementary Fig. 3). However, significant decreases in *Cnp* expression were observed on P12 at *bregma* +0.48 mm (p = 0.011) and *bregma* −3.3 mm (p = 0.034, Fig. 3A and B), indicating a reduction in oligodendrocyte maturation. To confirm this, the number of total (OLIG2+) and mature oligodendrocytes (CC1+/OLIG2+, Fig. 3C and D) was quantified in the cortex at P20. Less mature oligodendrocytes were observed in FIPH pups compared to controls in layer I-IV of the mediolateral cortex at *bregma* +0.48 mm (p = 0.005, Fig. 3E), and in layer V-VI of the mediolateral and lateral cortex at *bregma* −3.3 mm (p = 0.037 and p = 0.003, respectively, Fig. 3H), but not in the other locations of interest (p > 0.05, Fig. 3F and G). No differences in the total number of oligodendrocyte-lineage cells (OLIG2+) were observed in any of the examined regions (Supplementary Figs. 3C–F), indicating that the FIPH procedure induced a maturational arrest of oligodendrocytes rather than oligodendrocyte cell death in several cortical regions.

### 3.4 Fetal inflammation and postnatal hypoxia induce altered interneuron development

**Fig. 4:**
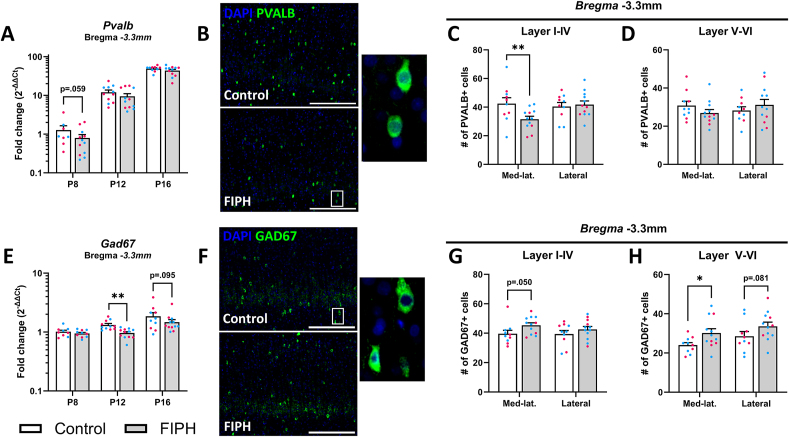
**Interneuron development was significantly altered in FIPH pups compared to controls.** (A) No significant difference in Pvalb gene expression was observed at bregma -3.33mm in FIPH rats compared to controls. (B) Representative images of PVALB+ cells in the mediolateral cortex. At P20, the number of PVALB+ cells was reduced in cortical layers I-IV of FIPH animals at bregma -3.3mm (C) but not in layers V-VI (D). (E) Gad67 gene expression is decreased during development at bregma -3.3mm at P12. F) Representative images of GAD67+ cells in the mediolateral cortex. The number of GAD67+ cells was significantly increased in the layers V-VI of the mediolateral cortex (H), but no significant differences were detected in layers I-IV of the cortex (G) or in layers V-VI of the lateral cortex (H) in FIPH animals compared to controls at bregma -3.3mm. Data is presented as mean ± SEM. For the individual data points, magenta circles represent female and blue circles male rats. Scalebar = 300μm, * p < .05, ** p < .01. Gene expression data is plotted on a log10-scale. (For interpretation of the references to color in this figure legend, the reader is referred to the Web version of this article.)

Next, we examined whether interneuron development was affected by the double-hit FIPH model. Over the course of postnatal cortical development, no significant differences in *Pvalb* gene expression were found at *bregma* +0.48 mm or *bregma* −3.3 mm (Fig. 4A, Supplementary Fig. 4A). Since gene expression in the whole cortex (see Fig. 2B) is only partially indicative of the number of cells, we next quantified the number of PVALB+ cells in the cortex at specific locations. A decrease in the number of PVALB+ cells was observed (Fig. 4B) in FIPH animals compared to controls in layers I-IV of the mediolateral cortex at *bregma* −3.3 mm (p = 0.009, Fig. 4C) but not at *bregma* +0.48 (p = 0.092, Supplementary Fig. 4B). No differences were found in layer V-VI at either location (Fig. 4D, Supplementary Fig. 4C).

To examine whether the reduction in number of PVALB+ cells in the mediolateral cortex coincided with a total decrease in interneurons, we next examined GAD67 as a marker for the total interneuron population. Although *Gad67* gene expression was decreased in FIPH animals compared to controls at P12 (p = 0.005) in FIPH animals compared to controls, the number of GAD67+ cells (Fig. 4F) in the mediolateral cortex was increased in layers V-VI (p = 0.036, Fig. 4H). No significant changes in the number of GAD67+ cells or *Gad67* gene expression were found at *bregma* +0.48 mm (Supplementary figure D-F). Together, these data indicate imbalances in cortical interneurons in FIPH animals in specific cortical locations.

### 3.5 Fetal inflammation at E20 and postnatal hypoxia leads to sustained immune activation

**Fig. 5:**
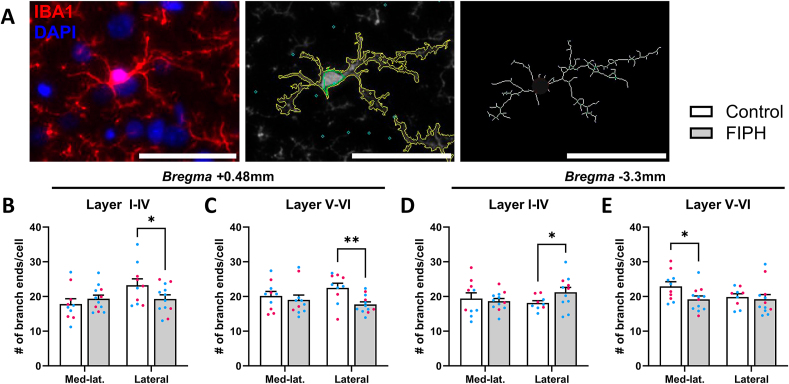
**Microglial morphology was significantly altered in FIPH pups compared to controls.** A) Example image of IBA1+/DAPI+ cell (first inset). IBA1+/DAPI+ soma were automatically detected (second inset, green) and used as the starting point to trace IBA1+ cell outline (yellow). Outlines are skeletonized to count the number of branch endpoints per cell (third inset). IBA1+ branches per cell were significantly altered in FIPH animals compared to controls in several cortical areas at bregma +0.48mm (B-C) and -3.3mm (D-E) in CTIP2+ (V-VI) and CTIP2- (I-IV) layers. For the individual data points, magenta circles represent female and blue circles male rats. Scalebar = 50 μm. Data is presented as mean ± SEM. * p < .05, ** p < .01. (For interpretation of the references to color in this figure legend, the reader is referred to the Web version of this article.)

To explore whether neuroinflammation was sustained at P20, IBA1+ cell count and morphology was examined at P20 (Fig. 5A). Although the total cortical number of microglia was similar between FIPH and control (data not shown), the number of branch endpoints was altered in layers I-IV and V-VI of the lateral cortex at *bregma* +0.48 mm (p = 0.043 and p = 0.005, respectively, Fig. 5B–C), and in layers V-VI of the mediolateral cortex and layers I-IV of the lateral cortex at *bregma* −3.3 mm (p = 0.026 and p = 0.030, respectively, Fig. 5D–E) after FIPH injury, suggesting microglia morphology in the cortex had not yet normalized more than two weeks after the second hit.

**Fig. 6:**
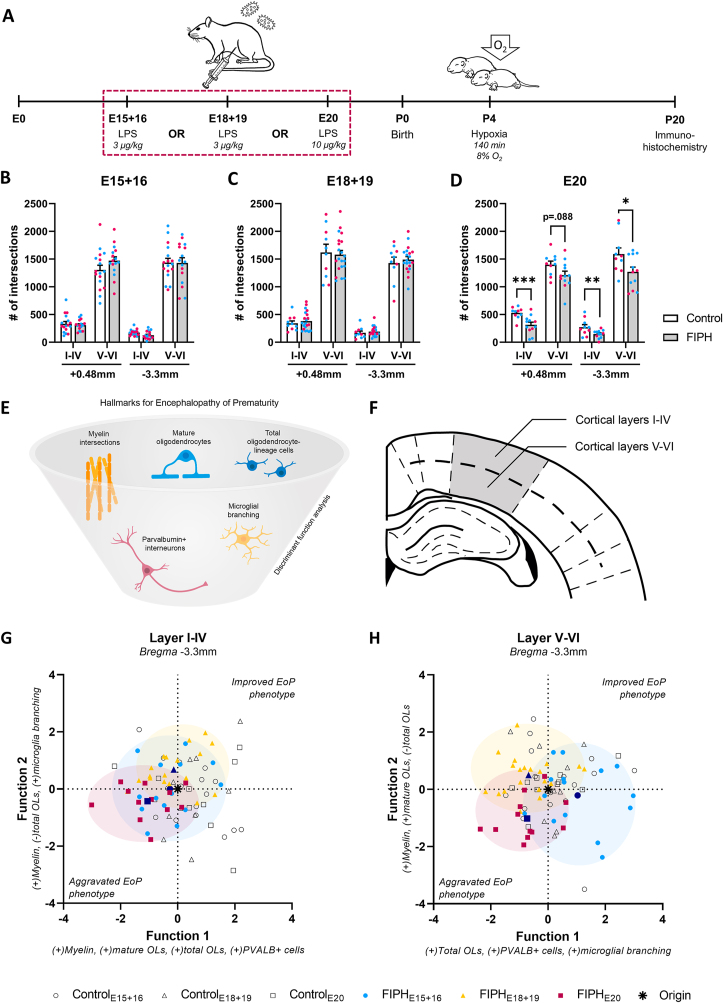
**(previous page). Timing of inflammation during gestation determines the EoP phenotype in the cortex**. A) FIPH and control pups were treated with lipopolysaccharide (LPS) or saline on embryonic day (E) 15+16, E18+19 or E20, respectively, and subjected to hypoxia at postnatal day (P) 4. Brains were extracted for immunohistochemistry at P20. Inflammation at E15+16, E18+19 or E20 combined with postnatal hypoxia led to no significant differences (B, C) or hypomyelination (D), respectively, in FIPH pups compared to controls in several cortical regions. For the individual data points, magenta circles represent female and blue circles male rats (B-D). (E) Discriminant function analysis (DFA) was performed to discriminate experimental paradigms on several markers representative for EoP. (F) DFA was performed separately for layers I-IV or V-VI in the mediolateral cortex. FIPH_E20_ rats displayed the most aggravated EoP phenotype in both layers I-IV (G) and V-VI (H) of the mediolateral cortex. E = embryonic day, P = postnatal day, LPS = lipopolysaccharide, (B-D) data is presented as mean ± SEM. * p < .05, ** p < .01, *** p < .001, _✲_ = centroid value for control animals. (For interpretation of the references to color in this figure legend, the reader is referred to the Web version of this article.)

### 3.6 Timing of fetal inflammation determines the severity of EoP hallmarks in the cortex

Finally, in order to investigate whether the timepoint of inflammation during gestation determines the severity of EoP pathology, cortical myelin expression was examined in offspring of dams injected with LPS at E15+16 (Fortier et al., 2007; Cui et al., 2009), E18+19 (Van Tilborg et al., 2018) and E20 (this paper), that were subsequently exposed to postnatal hypoxia at P4 (Fig. 6A). To establish a model with at least 75% litter survival, a dose of 3 μg/kg LPS was injected at E15+16 and E18+19 (as previously reported), while a dose of 10 μg/kg LPS was injected at E20. In the current study, dosages above 3 μg/kg (for E15+16 and E18+19) and 10 μg/kg (for E20) led to smaller litters, a higher proportion of miscarriages and in some cases, dam mortality (see Supplementary Fig. 1). No differences in myelin microstructure between FIPH and controls were observed in the mediolateral cortex when inflammation was induced at E15+16 (Fig. 6B) or E18+19 (Fig. 6C). As shown in Fig. 2P–S and (for consistency) in Fig. 6D, inflammation at E20 combined with postnatal hypoxia (i.e. FIPH_E20_) led to a decrease in myelin microstructure at in the mediolateral cortex at both examined *bregma* levels and most cortical locations.

To confirm whether FIPH_E20_ induced the most reliable EoP phenotype, we examined a broader presentation of EoP hallmarks using all model paradigms. To this aim, a multivariate analysis was performed containing five hallmarks indicative for EoP in the mediolateral cortex (myelin intersections, total oligodendrocyte-lineage cells, mature oligodendrocytes, PVALB+ interneurons, and microglial branching, Fig. 6E–F). A significant multivariate difference between experimental groups was observed in layers I-IV at *bregma* −3.3 mm (p = 0.040). Pairwise comparisons showed that FIPH_E20_ animals were most negatively affected compared to their respective controls (p = 0.028), which indicates an aggravated EoP-score in layers I-IV at *bregma* −3.3 mm in FIPH_E20_ animals, while FIPH_E15+16_ and FIPH_E18+19_ were not significantly different to controls in multivariate space (p = 0.138 and p = 0.195, respectively). MANOVA was followed up by a discriminant function analysis (DFA) to explore underlying factors that discriminated the experimental groups. The DFA revealed four discriminant functions of which the first was moderately correlated (*r* > 0.40) to the number of myelin intersections, the number of oligodendrocytes and PVALB+ interneurons, explaining 56.0% of the variance (Fig. 6G–Table 1), suggesting these factors contributed most to the EoP-phenotype in layers I-IV.

**Table 1:** Structure matrices showing correlations (*r*) of discriminant functions to underlying variables.

	*Bregma* −3.3 mm			
	I-IV		V-VI	
	DF1	DF2	DF1	DF2
Number of myelin intersections	0**.65**	0**.66**	0.17	0**.74**
Mature oligodendrocytes	0.35	−0.05	0.11	0**.44**
Total oligodendrocytes	0**.43**	−0.37	0**.55**	**−**0**.43**
Parvalbumin interneurons	0**.68**	−0.03	0**.61**	0.08
Microglia branch endpoints	−0.06	0**.65**	0**.56**	0.29

*Variance explained (%)*	*56.0*	*26.8*	*57.9*	*33.1*
*Wilk's Λ*	0*.59*	0*.78*	0*.53*	0*.75*
*p-value*	*	*n.s.*	**	*n.s.*

DF1 = discriminant function 1, DF2 = discriminant function 2. Correlations of r > 0.40 are in bold. *p < 0.05, **p < 0.01, n.s. = not significant. Wilk's Λ tests the hypothesis that all functions combined explain the variance within the population. For function 2, function 1 is omitted from this analysis, therefore remaining functions without function 1 do not significantly explain the variance.

In cortical layers V-VI at *bregma* −3.3 mm, a significant multivariate difference was observed between experimental groups (p = 0.005). Pairwise comparisons showed a trending effect of FIPH_E20_ animals compared to their relative controls in multivariate space (p = 0.059, Fig. 6H), which suggests subtle EoP-like pathology in layers V-VI at *bregma* −3.3 mm in FIPH_E20_ animals. FIPH_E15+16_ and FIPH_E18+19_ animals were not significantly different to their respective controls in multivariate space (p = 0.177 and p = 0.280, respectively). The DFA revealed four functions, of which the first correlated moderately to the total number of oligodendrocytes and PVALB+ interneurons, and to the number of microglia endpoints, explaining 57.9% of the variance (Table 1), suggesting these factors contributed to EoP-like pathology in layers V-VI.

At *bregma* +0.48 mm, layers I-IV, no significant differences were found in multivariate space (p = 0.181), therefore DFA was not performed. At the same *bregma* level in cortical layers V-VI, a significant multivariate difference was observed between experimental groups (p = 0.016). FIPH_E15+16_ animals deviated significantly from their respective controls (p < 0.001), showing a phenotype opposite to EoP (Supplementary Fig. 6), while FIPH_E18+19_ and FIPH_E20_ animals did not differ significantly from controls in layers V-VI at *bregma* +0.48 mm (p = 0.926 and p = 0.610, respectively). DFA revealed three discriminant functions (Supplementary Table 7). Immunohistochemical data of all examined cortical regions in FIPH_E15+16_ and FIPH_E18+19_ rats can be found in Supplementary Figs. 7 and 8, respectively. Together, the data presented here indicate that timing of inflammation is crucial to induce hallmarks of EoP, with fetal inflammation at E20 showing the most aggravated EoP phenotype.

## 4 Discussion

Brain injury resulting from preterm birth (EoP) is a key problem in global perinatal medicine, for which despite the major advancements in neonatal care, no curative treatments are currently available, and which can lead to lifelong cognitive, social and motor impairments (e.g. Duncan and Matthews, 2018; McGowan and Vohr, 2019). The development of clinically relevant animal models to uncover the underlying pathology of EoP and to explore and validate novel treatments is urgently needed to advance the field (Jantzie and Robinson, 2015; Kinney and Volpe, 2012; Ophelders et al., 2020). At present, there are few animal models available that mimic the more subtle and diffuse patterning of developmental preterm brain injury, without macrostructural changes, that is observed in the majority of current EoP patients (Ophelders et al., 2020). To reflect the multifaceted pathology in preterm infants, it is important that experimental models emulate the multifactorial etiology of EoP, including cerebral inflammation and oxygen imbalances. The FIPH model incorporates these as two temporally distinct hits, during clinically relevant stages of brain development with respect to the (extremely to very) preterm infant (Workman et al., 2013; Zeiss, 2021). To investigate whether future treatment strategies are effective for EoP regardless of instigating pathology, or if therapeutic efficacy is dependent on the cause of injury, it is crucial to develop different EoP models induced by various clinically relevant hits, such as intrauterine growth restriction, multiple inflammatory and/or hypoxic hits, and intraventricular hemorrhage. In this study, we performed an extensive behavioral, molecular and anatomical characterization of a double-hit rat model encompassing fetal inflammation at E20 and postnatal hypoxia at P4 in cortical regions known to be affected in EoP in both humans and animal models (Haynes et al., 2013; Mordel et al., 2016; Vaes et al., 2020/2021b). In addition, we investigated the effect of timing of maternal inflammation on EoP hallmarks in the cortex, providing insights into the heterogenous nature of EoP.

### 4.1 Behavioral deficits in FIPH rats are reminiscent of neurodevelopmental consequences of EoP

Preterm-born infants have an increased risk of persistent cognitive and motor impairments, and neurodevelopmental disorders, such as autism spectrum disorder (ASD) and attention deficit/hyperactivity disorder (ADHD) in adolescence and adulthood (Groeschel et al., 2019; Johnson et al., 2010; Johnson and Marlow, 2011; Meng et al., 2016; Neil and Volpe, 2018; Nosarti et al., 2008). To examine behavioral abnormalities in the FIPH model across the developmental, cognitive, (sensori)motor and social domains, a test battery was performed. Here, we show that FIPH pups already displayed neurodevelopmental impairments before weaning; they have more difficulty locating the nest, and emit less ultrasonic vocalizations upon maternal separation. Performance in the nest-seeking task is dependent on several factors, including motor and olfactory development, and social recognition memory, which has been shown to be impaired in rodent models of autism and prenatal methamphetamine or LPS exposure (Schneider and Przewłocki, 2005; Pometlová et al., 2009; Baharnoori et al., 2012). Impairments in emission of ultrasonic vocalizations in pups has previously been shown in models of maternal immune activation and autism (e.g. Baharnoori et al., 2012; Kirsten et al., 2015; Potasiewicz et al., 2020) and may reflect social and language impairments reported in children born preterm (Duncan and Matthews, 2018). Including these easy-to-implement behavioral tasks at pre-weaning age in preclinical studies may give an important indication of the effectiveness of potential early therapies in improving neurobehavioral outcome.

During adolescence, FIPH animals showed persistent behavioral alterations compared to controls. Juvenile FIPH animals showed a reduced interest in social interaction with an unfamiliar rat, which, combined with the reduced number of ultrasonic vocalizations, may reflect an aberrant social phenotype that could be linked to an increased risk of developing ASD in preterm infants (Crump et al., 2021). Furthermore, FIPH rats exhibited more anxiety-like behavior in the open field test (e.g. Gould et al., 2009; Seibenhener and Wooten, 2015), which may reflect the increased risk of developing anxiety-like symptoms in adolescents that were born very preterm (Sømhovd et al., 2012). Gross alterations in motor performance and/or hyperactivity were not detected during any of the behavioral tasks in FIPH animals. Rather, FIPH rats displayed increased sensitivity to benign tactile stimuli, which has also been observed in babies born preterm (26–35 weeks GA; André et al., 2020; Slater et al., 2010). Interestingly, these authors linked increased sensitivity in part to a higher exposure to (noxious) sensory stimuli earlier in life. Our results suggest that sensory processing as such might be altered in the FIPH model independent of early life sensory experiences, as these were consistent between the FIPH and control group. Atypical sensory processing has also been linked to ASD (e.g. Hadad and Yashar, 2022) and may be caused by a disbalance in excitatory and inhibitory neurons in the somatosensory cortex (Deemyad et al., 2022), which might also be manifested in the FIPH model (see section 4.2). Finally, FIPH animals displayed a deficit in spatial working memory (e.g. Wenk, 2001; Deacon and Rawlins, 2006), which is consistent with previous findings in a mouse model of MIA (Schaafsma et al., 2017) and neonatal diffuse white matter injury (dWMI; Vaes et al., 2021a), but not in a previous study performed by our group where 100 μg/kg LPS was injected at E18+19, followed by postnatal hypoxia (Van Tilborg et al., 2018). Therefore, the FIPH_E20_ model seems ideally suited to study spatial working memory deficits after preterm birth (Jongbloed-Pereboom et al., 2012).

Over the past decades, a clinical decline of (severe) motor deficits in preterm infants has been observed, while the incidence of non-motor neurodevelopmental deficits across the behavioral, cognitive, and social domains have become the more prominent correlates of EoP (Neil and Volpe, 2018). The FIPH model presented here therefore gives a valuable representation of the neurodevelopmental adversities that preterm-born infants nowadays encounter, including reduced sociability, increased anxiety, enhanced sensitivity, and working memory deficits, making it a suitable model to explore the long-term efficacy of potential therapies on functional outcome. Given the increased risk of developing ADHD in preterm infants, exploring attention and/or impulsivity could provide a potential avenue for further characterization of this model (e.g. Achterberg et al., 2022).

### 4.2 Inflammation at E20 and postnatal hypoxia cause pathophysiological hallmarks of EoP

Besides functional deficits, FIPH animals were examined for anatomical and molecular hallmarks of EoP at multiple locations in cerebral cortex, using both gene expression and immunohistochemical analyses. In FIPH animals, gene expression of myelin- and oligodendrocyte-associated genes is specifically impaired up to P12 which was associated with hypomyelination at P20. A decrease in global cortical myelin and loss of microstructural complexity was observed in FIPH animals, emulating a dWMI phenotype that is observed in preterm infants on clinical diffusion tensor imaging scans (Back and Miller, 2014). This maldevelopment of myelin coincided with a lower ratio of mature oligodendrocytes in several cortical areas, suggesting that alterations in myelin are preceded by hampered oligodendrocyte maturation (Van Tilborg et al., 2016). However, myelin deficits in other regions did not perfectly correspond to a significant local reduction in mature oligodendrocytes, suggesting that even with an almost equal number of mature myelin-producing oligodendrocytes, myelin production per cell or myelin quality may also have been directly impaired by the double-hit model. Indeed, others have established through electron microscopy that myelin sheath enwrapping is compromised after EoP-like injury (Ritter et al., 2013; Scheuer et al., 2015; Wang et al., 2021). Moreover, the number of internodes per oligodendrocyte and internode length could be altered (Barateiro et al., 2013; Bergles and Richardson, 2015). We speculate that besides a deficit in oligodendrocyte maturation, a mature oligodendrocyte population with reduced myelination capacity may have emerged after initial injury, thereby contributing to dWMI. Future tracing and birth-dating experiments could explore the dynamics of oligodendrocyte development after neonatal brain injury, and their ability to mature and myelinate the cortex effectively.

To examine whether the FIPH paradigm can be used to study EoP-related interneuron deficits, we examined the number of cortical GAD67+ and PVALB+ interneurons and interneuron gene expression. Interestingly, we found a local decrease in the number of PVALB+ interneurons and a widespread increase in GAD67+ interneurons at P20 in animals after FIPH, which is similar to what was previously observed in a double-hit model of EoP (Vaes et al., 2020). Likewise, a reduction in PVALB+ interneurons has previously been found in *post mortem* tissue from extremely preterm infants (Panda et al., 2018) and in other animal models of EoP (Volpe, 2009; Buser et al., 2012; Back and Miller, 2014; Stolp et al., 2019). PVALB+ cells are fast-spiking interneurons that are particularly vulnerable to preterm birth-related factors, such as oxidative stress (Moore et al., 2018), excess glutamate (Vaes et al., 2020; Desfeux et al., 2010; Roux et al., 2015), and hypomyelination (Lim et al., 2018; Hafner et al., 2019; Wamsley and Fishell, 2017), due to their energy requirements, excitatory inputs and developmental trajectory (Zhang et al., 2016; Ruden et al., 2021; Juarez and Martínez-Cerdeño, 2022). Moreover, we speculate that the cerebral milieu after FIPH, containing factors such as excess glutamate and chemokines, could have affected interneuron migration, leading to an altered distribution of interneuron subtypes, like PVALB+ cells, in the cortex (López-Bendito et al., 2008; Léger et al., 2020; Toudji et al., 2023). In addition, it has been demonstrated that preterm birth in rabbits induced proliferation and population of interneuron progenitors in the medial ganglionic eminence (Tibrewal et al., 2018) which could explain the increase in GAD67+ cell numbers after FIPH. Although our data show that there were more cells expressing cytosolic GAD67+ present at P20, the reduction in cortical *Gad67* gene expression during development in FIPH animals may indicate reduced GABAergic capacity within these cells (Brown et al., 2015; Lau and Murthy, 2012). Decreased GAD67 protein expression has previously been found after neonatal LPS exposure (Wu et al., 2022) and oxidative stress (Scheuer et al., 2022). Alternatively, this discrepancy between lower *Gad67* gene expression and more cytosolic GAD67+ cells may be explained by the reduced spatial resolution of tissue dissection for gene expression analysis versus local histology, as the first may overlook local increases or decreases. Future research could examine the relative contributions of other interneuron subsets (SST+ or 5HT3aR+) within the abundant GAD67+ cell population in the FIPH model (e.g. Duchatel et al., 2019), to establish whether these subtypes might be overrepresented in this model. While more research is needed on the origin of interneuron imbalances in the FIPH model, our findings indicate this model is highly clinically relevant. Alterations in the number of interneurons may disturb the excitatory/inhibitory balance in the neuronal network, which has been linked to several neurodevelopmental disorders associated with preterm birth, such as ASD and ADHD (Hashemi et al., 2018; Scheuer et al., 2021; Jendryka et al., 2023; Johnson and Marlow, 2011; Juarez and Martínez-Cerdeño, 2022). We speculate that the interneuron aberrations in the FIPH model may be linked to altered sensory processing and deficits in social behaviors and working memory reported here (Brown et al., 2015; Lacaille et al., 2019).

Microglia have been implicated as important contributors to the pathophysiology of EoP (Van Tilborg et al., 2016; Volpe, 2019; Fleiss et al., 2021). Activation of microglia by pre- or postnatal infections and/or oxygen imbalances triggers the release of pro-inflammatory cytokines and free radicals that are detrimental to oligodendrocyte and interneuron precursor cells, while also impeding the trophic function of microglia during normal brain development (e.g. Volpe, 2019; Vaes et al., 2021b; Fleiss et al., 2021). Although microglial morphology in the cortex was subtly altered in the FIPH model up to P20, it is likely that major inflammatory processes have largely been resolved at this time. While it is possible that microglia present with an activated phenotype for a prolonged period of time (Fleiss et al., 2021), morphological analysis may not be sufficient to unravel dynamic microglial activation states present during the tertiary phase (Fleiss and Gressens, 2012), which could be addressed in future studies using more powerful transcriptomic or proteomic techniques in the FIPH model (e.g. Klein et al., 2022; Paolicelli et al., 2022).

Together, our results demonstrate that the FIPH model is relevant to study the dynamics of immune activation, maturational arrest of oligodendrocytes, hypomyelination, and interneuron deficits in EoP, and may be used to establish the efficacy of potential therapies.

### 4.3 Timing of inflammation during gestation determines the severity and pattern of EoP pathology

Preterm birth can occur at several different stages of pregnancy, with maternal inflammation being one of the major risk factors inducing preterm labor (Daskalakis et al., 2023). Here, we examined whether the timepoint of inflammation during gestation affects the severity and pattern of EoP pathology in the double-hit FIPH rat model. We found that FIPH_E20_ caused the most severe EoP phenotype in the cerebral cortex, which could be attributed to the shortened duration between the first and second hit, leading to the accumulation of detrimental processes such as the release of free radicals (Khwaja and Volpe, 2008; Moore et al., 2018). Conflicting evidence suggests that preconditioning with LPS may be either protective or detrimental to later hypoxic(-ischemic) brain injury, therefore the relative timing of our hits is likely one of the determining factors for the severity of injury (Hagberg et al., 2015; Eklind et al., 2005; Hickey et al., 2011; Dhillon et al., 2015; Lu et al., 2023). Alternatively, the different LPS dosages that were used to obtain viable litters at different injection timepoints (see section 2.3 and Supplementary Fig. 1) could have contributed to differences in EoP-like pathology. In particular, the usage of a significantly lower dose and different LPS manufacturer than was previously utilized by our group (Van Tilborg et al., 2018) may have led to the absence of a clear WMI-phenotype in the FIPH_E18+19_ model reported here.

The severity of EoP pathology may also be determined by the stage of fetal brain development when inflammation occurs. Interestingly, FIPH_E15+16_ animals had a denser myelin microstructure compared to healthy controls in the cortex rostrally, which was accompanied by an increase in both total and mature oligodendrocytes (Supplementary Fig. 7). During development, oligodendrocytes migrate in three waves from the ganglionic eminences, with the third, dorsal wave eventually becoming the dominant population in the cerebral cortex that gives rise to mature, myelinating oligodendrocytes (Kessaris et al., 2006). In rodents, the second and third migration waves take place from E15.5 and E21/P0, respectively (reviewed by Van Tilborg et al., 2018). Therefore, inflammation occurring at E15+16 may interfere with oligodendrocyte migration leading to regional imbalances, whereas inflammation at E20 might specifically affect the third wave of oligodendrocytes resulting in lasting effects of hypomyelination in the cortex. Similarly, PVALB+ interneurons are produced in waves and tangentially migrate towards the cortex right until birth in rodents, after which laminar distribution continues throughout the first postnatal week following an “inside-out” trajectory (Lim et al., 2018). Insults occurring at different stages during this process may have led to specific regional interneuron imbalances, such as a caudal increase of PVALB+ interneurons in FIPH_E15+16_ animals, and local rostral or caudal decreases of PVALB+ cells found in the FIPH_E18+19_ and FIPH_E20_ models, respectively.

Together, these data indicate that gestational age at the time of an inflammatory insult may profoundly impact the developmental programs of oligodendrocytes, myelination, and interneurons in the preterm brain. Transcriptomic analyses, tissue clearing and/or fate mapping techniques could provide more insight into the complex mechanisms by which EoP-related insults interfere with normal cortical development. Moreover, this study provides important indications to clinically examine whether the timing of exposure of the human fetal brain to inflammatory episodes during pregnancy predicts the risk of developing EoP.

### 4.4 Limitations

Although this study gives an extensive overview of behavioral and anatomical hallmarks of double-hit EoP model(s), it has several limitations. Firstly, in this study we have predominantly focused on EoP hallmarks in *cortical* areas of the brain. The period in which preterm infants are at the highest risk to develop EoP (up to 32 weeks of gestational age) coincides with various dynamic cellular events that shape the developing cortex, while interruptions of these processes predispose the cortex to atypical network development and potential long-term functional consequences (Volpe, 2019; Thompson et al., 2007; Dimitrova et al., 2021; Wang et al., 2023). Although two coronal levels were examined to account for spatiotemporal differences in the developing brain, it is conceivable that the FIPH model may have differentially impacted other brain regions, not examined in this study. Indeed, EoP in patients is characterized by widespread (diffuse) white and gray matter injury across numerous brain structures, such as the posterior limb of the internal capsule, the thalamus, hippocampus, and the cerebellum (Hinojosa-Rodríguez et al., 2017), which are unexplored in the present study. Of note, the significance of cerebellar injury due to prematurity is increasingly recognized and could be investigated further in the FIPH model (Klein et al., 2022; Limperopoulos et al., 2014; Wu et al., 2020). Furthermore, interneuron imbalances have also been found in other brain regions in preterm post-mortem tissue and other preclinical EoP models, e.g. in the prefrontal cortex (Lacaille et al., 2019) and hippocampus (Vaes et al., 2020). Therefore, behavioral deficits reported in this model may only be partly attributed to the cortical alterations reported here. Furthermore, we have studied gene expression and structural deficits in the cortex *up to P20*. Previous studies by our group have shown that cortical myelin and interneuron deficits largely restored over time in rodents, even though functional deficits persisted (Van Tilborg et al., 2018; Vaes et al., 2020, 2021a). We hypothesize that spontaneous recovery of examined cortical regions might similarly occur in the current model. Potentially, further investigation into different brain structures could indicate why functional differences persist while the cortical alterations potentially do not.

Secondly, the different fetal inflammation timing paradigms have been tested in separate rat cohorts, which was resolved here by normalizing FIPH animals to their relative control group (i.e. NaCl injection at the concomitant gestational days within the same cohort). To further disentangle the role of immune activation at different stages during prenatal development on oligodendrocyte and interneuron development, these conditions might be compared within a single experimental cohort. This may provide important insights into the clinical diversity of EoP and initiate the development of personalized treatments based on clinical events relative to gestational age.

Finally, although both male and female offspring was included, this study was not powered to detect potential differences between sexes (see Supplementary Table 1 for the sex distribution per group). Although preterm birth occurs in both sexes, the incidence is higher for boys (e.g. Peelen et al., 2016), who are typically also more susceptible to adverse neurodevelopmental outcomes after preterm birth (e.g. Duncan and Matthews, 2018; Ardalan et al., 2019). Preliminary analyses in our dataset did not show interactions between sex and experimental group, however, studies with increased statistical power may be able to distinguish a potentially more severe EoP-phenotype in the male FIPH group.

## 5 Conclusion

In conclusion, a double-hit model encompassing timed fetal inflammation at E20 and postnatal hypoxia at P4 generates a clear EoP phenotype with anatomical hallmarks of EoP in the cortex, corresponding to developmental, social, sensory, and cognitive aberrations. In future, this double-hit model could be used to investigate pathophysiological mechanisms and potential therapies for EoP, such as mesenchymal stem cells and nutrition-based therapies.

## Funding

This project has received funding from the European Union's Horizon 2020 Research and Innovation programme under Grant Agreement #874721 (PREMSTEM). This work was supported by the Dutch Research Council (NWO; ZonMw Program “More Knowledge with Fewer Animals”). Funding agencies were not involved in the study design; collection, analysis and interpretation of data; the writing of the report; nor in the decision to submit the article for publication.

## CRediT authorship contribution statement

**M.J.V. Brandt:** Conceptualization, Data curation, Formal analysis, Funding acquisition, Investigation, Methodology, Project administration, Software, Validation, Visualization, Writing – original draft, Writing – review & editing. **C.M. Kosmeijer:** Conceptualization, Data curation, Formal analysis, Funding acquisition, Investigation, Methodology, Project administration, Validation, Visualization, Writing – original draft, Writing – review & editing. **E.J.M. Achterberg:** Data curation, Formal analysis, Investigation, Methodology, Resources, Validation, Writing – review & editing. **C.G.M. de Theije:** Conceptualization, Funding acquisition, Methodology, Project administration, Resources, Validation, Writing – review & editing. **C.H. Nijboer:** Conceptualization, Funding acquisition, Methodology, Project administration, Resources, Supervision, Validation, Writing – review & editing.

## Declaration of competing interest

The authors declare that they have no known competing financial interests or personal relationships that could have appeared to influence the work reported in this paper.

## Data Availability

Data will be made available on request.
