# Supplementary material for: Timed fetal inflammation and postnatal hypoxia cause cortical white matter injury, interneuron imbalances, and behavioral deficits in a double-hit rat model of encephalopathy of prematurity
Source: Brain Behav Immun Health. 2024 Jul 5;40:100817. doi: 10.1016/j.bbih.2024.100817 (PMC11345510; doi:10.1016/j.bbih.2024.100817)
Supplement: Multimedia component 1 [file mmc1.pdf]

Supplementary materials

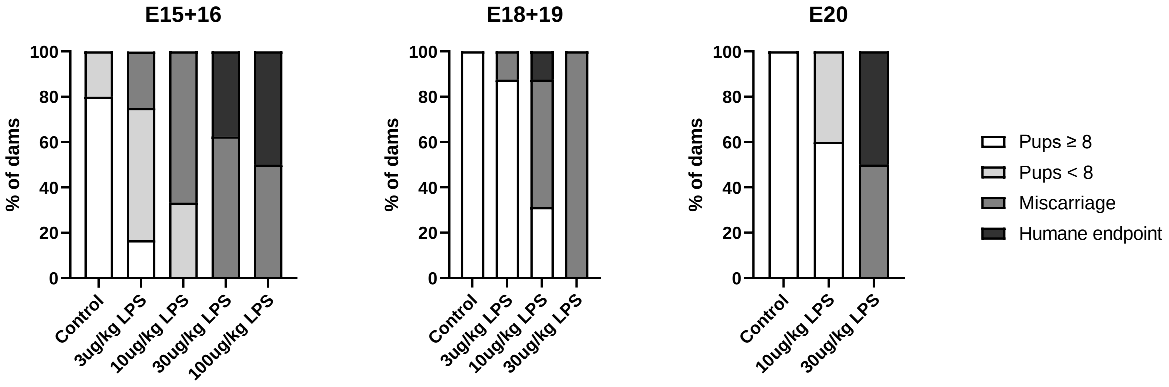

Supplementary figure 1. Litter and dam survival after LPS injection was affected by the LPS dose and embryonic day (E) of injection. *Litter survival in the NaCl-injected control groups was 100% throughout all fetal timepoints, while survival in the LPS groups differed based on LPS dosage and time point. Litter size was also affected by LPS injection, with higher dosages yielding smaller litters (< 8 pups). Pregnancy was classified as a miscarriage when none of the pups survived, due to preterm and/or still birth. The humane endpoint was reached when the dam did not recover from LPS within 24 hours (showing severe signs of illness). E15+16: control; n = 6, LPS; 3µg/kg, n = 12, 10µg/kg, n = 4, 30µg/kg, n = 8, 100µg/kg, n = 8, E18+19: control; n = 3, LPS; 3µg/kg, n = 8, 10µg/kg, n = 16, 30µg/kg, n = 6, E20: control; n = 2, LPS; 10µg/kg, n = 5, 30µg/kg, n = 2.*

Supplementary table 1. Overview of sample sizes used and allocation of males and females per group in this study.

	Control		FIPH	
Immunohistochemistry (P20)	Total	M | F	Total	M | F
$FIPH_{E15+16}$ model	17	9 | 8	16	8 | 8
$FIPH_{E18+19}$ model	10	6 | 4	21	12 | 9
$FIPH_{E20}$ model	10	5 | 5	12	8 | 4
Gene expression ($FIPH_{E20}$)	Total	M | F	Total	M | F
P8	9	4 | 5	12	6 | 6
P12	11	5 | 6	13	6 | 7
P16	10	5 | 5	12	6 | 6
Behavioral analysis ($FIPH_{E20}$)	Total	M | F	Total	M | F
Nest seeking[1]	20	10 | 10	21	12 | 9
Ultrasonic vocalizations[2]	24	12 | 12	24	10 | 14
Direct social interaction[2,3,#]	35	18 | 17	31	18 | 13
OF, ART, T-maze, beam walk[3]	43	22 | 21	34	23 | 11

[1,2,3] Separate behavioral cohorts 1, 2, 3. [#] Sample size is reported per experimental pair, OF = open field, ART = adhesive removal task, E = embryonic day, P = postnatal day, M = male, F = female

Supplementary table 2. Overview of the RT-qPCR primer sequences used in the study			
Gene name	*Symbol*	*Forward primer sequence*	*Reverse primer sequence*
Hypoxanthine phosphoribosyltransferase 1	Hprt1	TTCCTTGGTCAAGCAGTACAG	AACAAAGTCTGGCCTGTATCC
Phosphoglycerate kinase 1	Pgk1	AGATTACCTTGCCTGTTGACTT	CACAGCCTCAGCATATTTCTTAC
Myelin-associated glycoprotein	Mag	GCCTTCAACCTGTCTGTGGA	AGGGTTCCGGGTTGGATTTT
Myelin basic protein	Mbp	CCGAGGAGAGTGTGGGTTTA	TCTGGAGGGTTTGTTTCTGG
Myelin-associated oligodendrocyte basic protein	Mobp	GCCAGATGGGAGCTTGAAAAC	ATCTCACGCTTGGAGTTGAGG
Myelin oligodendrocyte glycoprotein	Mog	TCCGTGCAGAAGTCGAGAAT	CTTCGGTGCAGCCAGTTGTA
Proteolipid protein 1	Plp1	AGCCGAGTTCCAAATGACCT	AGATCAGAACTTGGTGCCTCG
Platelet derived growth factor alpha	Pdgfra	GCCTTACGACTCCAGATGGG	CCACTTTCCCAAATGCACCG
Oligodendrocyte transcription factor 2	Olig2	TGGATGCTTAACAGAGACCCG	CGGAGACGATCTAGGCTTTCG
2’,3’-cyclic nucleotide 3’ phophodiesterase	Cnp	CTGTGACTACGGGAAGGCCA	GCCGTAAGATCTCCTCACCA
Parvalbumin	Pvalb	GGGATGGCAAGATTGGGGTT	GGTGTCATTCGAGGGCCATA
Glutamate decarboxylase 67	Gad67	GCTCCCTGTGGCTGAATCG	TGTAGGACGCAGGTTGGTAG

Supplementary table 3. Results of the statistical analysis for behavioral tasks

		Main effects		*Interaction effect*
		Group*	PND**	Group x PND
Nest seeking#	*Fig. 1B*	U(39) = 124.0, p = .024<br>r = .351	-	-
Ultrasonic vocalizations	*Fig. 1C*	F(1,48) = 1.38, p = .247<br>r = .167##	F(2,95) = 10.47, p < .001<br>###	F(2,95) = 2.55, p = .083<br>###
Social engagement	*Fig. 1D*	t(64) = 2.79, p = .007<br>r = .329	-	-
Open field#	*Fig. 1E*	U(72) = 453.0, p = .031<br>r = .253	-	-
Adhesive removal#	*Fig. 1F*	U(73) = 383.0, p < .001<br>r = .377	-	-
T-maze#	*Fig. 1G*	U(75) = 356.5, p < .001<br>r = .438	-	-
Tapered beam walk#	*Fig. 1H*	U(75) = 620.0, p = .258<br>r = .130	-	-

** Between-subjects factor, ** within-subjects factor, # Mann-Whitney test was utilized due to non-normal data distribution, ## mixed model: effect size is calculated as $\sqrt{(\frac{F}{F+df})}$, in which df = degrees of freedom calculated using Satterthwaite method, ### effect size not provided for factors with more than two levels, PND = postnatal day.*

Supplementary table 4. Results of the statistical analysis for gene expression

		Main effects		*Interaction effect*
		Group*	PND*	Group x PND
Bregma +0.48mm				
Mbp	*Supp. fig. 2B*	F(1,42) = 7.07, p = .011<br>r = .380	F(1,42) = 22.81, p < .001<br>r = .593	F(1,42) = 6.90, p = .011<br>r = .378
Mag	*Supp. fig. 2C*	F(1,42) = 7.04, p = .011<br>r = .379	F(1,42) = 27.59, p < .001<br>r = .630	F(1,42) = .89, p = .349<br>r = .144
Mog	*Supp. fig. 2D*	F(1,42) = 9.50, p = .004<br>r = .429	F(1,42) = 54.58, p < .001<br>r = .752	F(1,42) = 5.26, p = .027<br>r = .333
Mobp	*Supp. fig. 2E*	F(1,42) = 10.19, p = .003<br>r = .442	F(1,42) = 46.75, p < .001<br>r = .726	F(1,42) = 6.18, p = .017<br>r = .358
Plp1	*Supp. fig. 2F*	F(1,41) = 13.67, p < .001<br>r = .500	F(1,41) = 44.60, p < .001<br>r = .722	F(1,41) = 7.21, p = .010<br>r = .387
Cnp	*Fig. 3A*	F(1,60) = 5.66, p = .021<br>r = .294	F(2,60) = 19.14, p < .001<br>[#]	F(2,60) = 1.51, p = .230<br>[#]
Pvalb	*Supp. fig. 4A*	F(1,42) = 1.55, p = .220<br>r = .189	F(1,42) = 90.13, p < .001<br>r = .826	F(1,42) = .14, p = .709<br>r = .058
Gad67	*Supp. fig. 4D*	F(1,40) = 1.01, p = .321<br>r = .157	F(1,40) = 55.08, p < .001<br>r = .761	F(1,40) = .014, p = .907<br>r = .019
Bregma -3.3mm				
Mbp	*Fig. 2C*	F(1,59) = 7.76, p = .007<br>r = .341	F(2,59) = 239.12, p < .001<br>[#]	F(2,59) = 3.08, p = .053<br>[#]
Mag	*Fig. 2D*	F(1,59) = 5.74, p = .020<br>r = .300	F(2,59) = 115.47, p < .001<br>[#]	F(2,59) = 2.76, p = .072<br>[#]
Mog	*Fig. 2E*	F(1,58) = 13.97, p < .001<br>r = .441	F(2,58) = 353.29, p < .001<br>[#]	F(2,58) = 3.59, p < .001<br>[#]
Mobp	*Fig. 2F*	F(1,58) = 9.07, p = .004<br>r = .368	F(2,58) = 239.91, p < .001<br>[#]	F(2,58) = 2.62, p = .081<br>[#]
Plp1	*Fig. 2G*	F(1,59) = 9.50, p = .003<br>r = .372	F(2,59) = 200.78, p < .001<br>[#]	F(2,59) = 2.18, p = .122<br>[#]
Cnp	*Fig. 3B*	F(1,59) = 2.92, p = .093<br>r = .217	F(2,59) = 45.68, p < .001<br>[#]	F(2,59) = 1.64, p = .204<br>[#]
Pdgfra	*Supp. fig. 3A*	F(1,59) = .31, p = .581<br>r = .072	F(2,59) = 230.01, p < .001<br>[#]	F(2,59) = .51, p = .603<br>[#]
Olig2	*Supp. fig. 3B*	F(1,59) = .08, p = .781<br>r = .036	F(2,59) = 124,94, p < .001<br>[#]	F(2,59) = .50, p = .612<br>[#]
Pvalb	*Fig. 4A*	F(1,59) = 5.01, p = .029<br>r = .280	F(2,59) = 286.88, p < .001<br>[#]	F(2,59) = .47, p = .625<br>[#]
Gad67	*Fig. 4E*	F(1,58) = 8.54, p = .005<br>r = .358	F(2,58) = 17.90, p < .001<br>[#]	F(2,58) = 1.27, p = .287<br>[#]

** Between-subjects factor, [#] effect size not provided for factors with more than two levels, effect size is calculated as* $\sqrt{(\frac{F}{F+df})}$*, PND = postnatal day.*

Supplementary table 5. Results of the statistical analysis for histological hallmarks of EoP of FIPH$_{E20}$

			Main effects		*Interaction effect*
			Group*	Region**	Group x Region
**% MBP+ area**					
+0.48mm	Layer I-IV	*Fig. 2I*	t(20) = 2.57, p = .018<br>r = .498	-	-
+0.48mm	Layer V-VI	*Fig. 2J*	t(20) = 1.13, p = .273<br>r = .244	-	-
-3.3mm	Layer I-IV	*Fig. 2K*	t(20) = 2.99, p = .007<br>r = .556	-	-
-3.3mm	Layer V-VI	*Fig. 2L*	t(20) = 3.31, p = .004<br>r = .595	-	-
**Myelin microstructure**					
+0.48mm	Layer I-IV	*Fig. 2P*	F(1,22) = 28.54, p < .001<br>r = .751[##]	F(1,22) = 6.22, p = .021<br>r = .469[##]	F(1,22) = .04, p = .842<br>r = .043[##]
+0.48mm	Layer V-VI	*Fig. 2Q*	F(1,22) = 7.21, p = .014<br>r = .497[##]	F(1,22) = 13.50, p = .001<br>r = .617[##]	F(1,22) = 3.38, p = .079<br>r = .365[##]
-3.3mm	Layer I-IV	*Fig. 2R*	F(1,22) = 10.09, p = .004<br>r = .561[##]	F(1,22) = 34.63, p < .001<br>r = .782[##]	F(1,22) = .09, p = .770<br>r = .064[##]
-3.3mm	Layer V-VI	*Fig. 2S*	F(1,22) = 6.45, p = .019<br>r = .476[##]	F(1,22) = 14.37, p = .001<br>r = .629[##]	F(1,22) = .01, p = .942<br>r = .021[##]
**Mature oligodendrocytes**					
+0.48mm	Layer I-IV	*Fig. 3D*	F(1,40) = 8.59, p = .006<br>r = .420[##]	F(1,40) = 5.82, p = .021<br>r = .356[##]	F(1,40) = 1.66, p = .205<br>r = .200[##]
+0.48mm	Layer V-VI	*Fig. 3E*	F(1,22) = 2.54, p = .125<br>r = .322[##]	F(1,22) = .23, p = .636<br>r = .102[##]	F(1,22) = .21, p = .651<br>r = .097[##]
-3.3mm	Layer I-IV	*Fig. 3F*	F(1,22) = 3.17, p = .089<br>r = .355[##]	F(1,22) = .01, p = .932<br>r = .021[##]	F(1,22) = .27, p = .606<br>r = .110[##]
-3.3mm	Layer V-VI	*Fig. 3G*	F(1,22) = 10.98, p = .003<br>r = .577[##]	F(1,22) = 3.64, p = .069<br>r = .377[##]	F(1,22) = .76, p = .393<br>r = .183[##]
**Total oligodendrocyte lineage cells**					
+0.48mm	Layer I-IV	*Supp. fig. 3C*	F(1,22) = .01, p = .944<br>r = .021[##]	F(1,22) = 14.34, p = .001<br>r = .628[##]	F(1,22) = .07, p = .793<br>r = .056[##]
+0.48mm	Layer V-VI	*Supp. fig. 3D*	F(1,22) = 1.04, p = .318<br>r = .212[##]	F(1,22) = .75, p = .395<br>r = .182[##]	F(1,22) = .27, p = .608<br>r = .110[##]
-3.3mm	Layer I-IV	*Supp. fig. 3E*	F(1,40) = 1.45, p = .236<br>r = .187[##]	F(1,40) = 130.61, p < .001<br>r = .875[##]	F(1,40) = .71, p = .403<br>r = .132[##]
-3.3mm	Layer V-VI	*Supp. fig. 3F*	F(1,22) = .06, p = .806<br>r = .052[##]	F(1,22) = 11.93, p = .002<br>r = .593[##]	F(1,22) = .289, p = .596<br>r = .114[##]
**Parvalbumin+ interneurons**					
+0.48mm	Layer I-IV	*Supp. fig. 4B*	F(1,22) = 1.61, p = .218<br>r = .261[##]	F(1,22) = .04, p = .846<br>r = .043[##]	F(1,22) = 1.57, p = .224<br>r = .258[##]
+0.48mm	Layer V-VI	*Supp. fig. 4C*	F(1,39) = .34, p = .566<br>r = .093[##]	F(1,39) = .05, p = .829<br>r = .036[##]	F(1,39) = .08, p = .777<br>r = .045[##]
-3.3mm	Layer I-IV	*Fig. 4C*	F(1,22) = 2.47, p = .131<br>r = .318[##]	F(1,22) = 2.68, p = .116<br>r = .330[##]	F(1,22) = 5.92, p = .024<br>r = .460[##]
-3.3mm	Layer V-VI	*Fig. 4D*	F(1,22) = .03, p = .856<br>r = .037[##]	F(1,22) = .19, p = .666<br>r = .093[##]	F(1,22) = 3.30, p = .083<br>r = .361[##]

GAD67+ interneurons

+0.48mm	Layer I-IV	*Supp. fig. 4E*	F(1,22) = .26, p = .613<br>r = .108##	F(1,22) = 2.75, p = .111<br>r = .333##	F(1,22) = .02, p = .901<br>r = .030##
	Layer V-VI	*Supp. fig. 4F*	F(1,40) = .00 , p = .971<br>r = .000##	F(1,40) = .08, p = .774<br>r = .045##	F(1,40) = 3.01, p = .090<br>r = .265##
-3.3mm	Layer I-IV	*Fig. 4G*	F(1,22) = 3.46, p = .076<br>r = .369##	F(1,22) = .94, p = .342<br>r = .202##	F(1,22) = .71, p = .410<br>r = .177##
	Layer V-VI	*Fig. 4H*	F(1,40) = 7.13, p = .014<br>r = .389##	F(1,40) = 4.34, p = .049<br>r = .313##	F(1,40) = .08, p = .78<br>r = .045##

IBA+ branch endpoints

+0.48mm	Layer I-IV	*Fig. 5E*	F(1,22) = .56, p = .461<br>r = .158##	F(1,22) = 7.94, p = .010<br>r = .515##	F(1,22) = 8.23, p = .009<br>r = .522##
	Layer V-VI	*Fig. 5F*	F(1,22) = 5.22, p = .032<br>r = .438##	F(1,22) = .35, p = .561<br>r = .125##	F(1,22) = 3.55, p = .073<br>r = .373##
-3.3mm	Layer I-IV	*Fig. 5G*	F(1,20) = 1.47, p = .239<br>r = .262##	F(1,20) = 1.20, p = .287<br>r = .238##	F(1,20) = 4.36, p = .050<br>r = .423##
	Layer V-VI	*Fig. 5H*	F(1,21) = 2.89, p = .104<br>r = .348##	F(1,20) = 2.74, p = .113<br>r = .347##	F(1,20) = 2.89, p = .105<br>r = .355##

** Between-subjects factor, ** within-subjects factor, # effect size not provided for factors with more than two levels, ## mixed model: effect size is calculated as $\sqrt{(\frac{F}{F+df})}$, in which df = degrees of freedom calculated using Satterthwaite method, PND = postnatal day.*

Supplementary table 6. Results of the multivariate analyses between model paradigms

		Pillai's Trace (V)	F	df	p
+0.48mm	Layer I-IV	.366	1.263	25,400	.181[#]
	Layer V-VI	.514	1.742	25,380	.016[#]
-3.3mm	Layer I-IV	.481	1.577	25,370	.040[#]
	Layer V-VI	.561	1.945	25,385	.005[#]

[#] effect size not provided for factors with more than two levels, PND = postnatal day.

Supplementary table 7. Structure matrices showing correlations (*r*) of discriminant functions to underlying variables

	Bregma +0.48mm	
	Layer V-VI	
	F1	F2
Number of myelin intersections	**.45**	**.50**
Mature oligodendrocytes	**.52**	.10
Total oligodendrocytes	**.64**	-.20
Parvalbumin interneurons	.26	**-.64**
Microglia branch endpoints	.14	.23
Variance explained (%)	*91.3*	*7.5*
Wilk's Λ	*.52*	*.93*
p-value	****	*n.s.*

*F1 = discriminant function 1, F2 = discriminant function 2. Correlations of r > .40 are in bold. **p < .01, n.s. = not significant. Wilk's Λ tests the hypothesis that all functions combined explain the variance within the population. For function 2, function 1 is omitted from this analysis, therefore remaining functions without function 1 do not significantly explain the variance.*

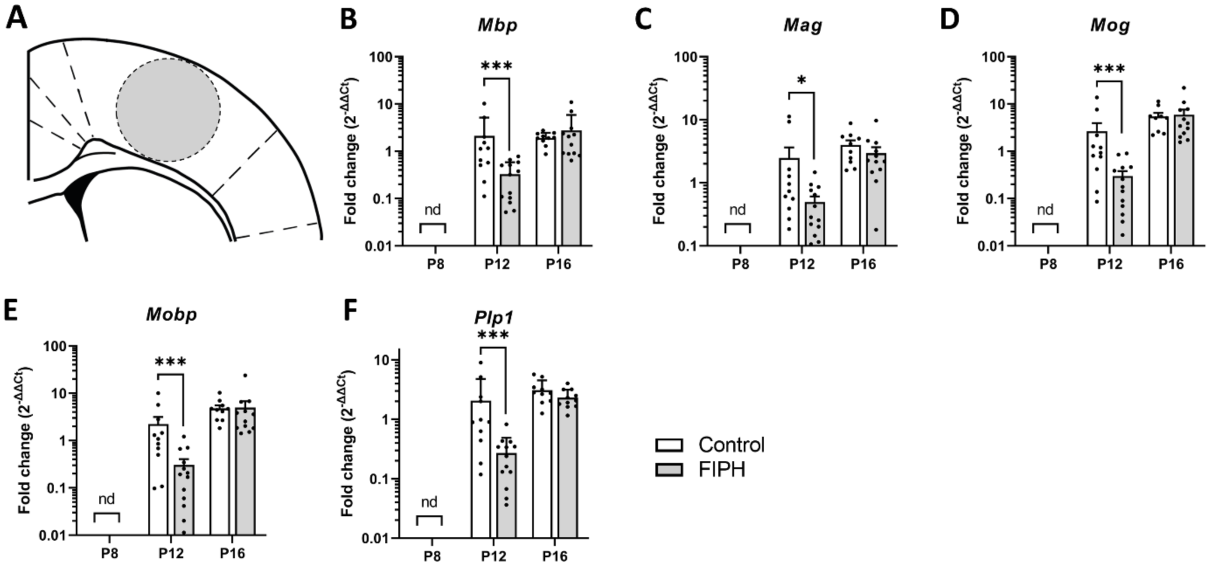

Supplementary figure 2. ***Rostral myelin gene expression is significantly impaired at P12 in FIPH animals compared to controls.*** *The expression of myelin-associated genes from cortical tissue punches (dashed outline) at bregma +0.48mm (A), was significantly lower at P12, but not at P16 in FIPH animals compared to controls (B-F). Data is presented as mean ± SEM and plotted on a log10-scale. * p < .05, *** p < .001. FIPH, fetal inflammation and postnatal hypoxia; nd = expression was not reliably detected; P = postnatal day.*

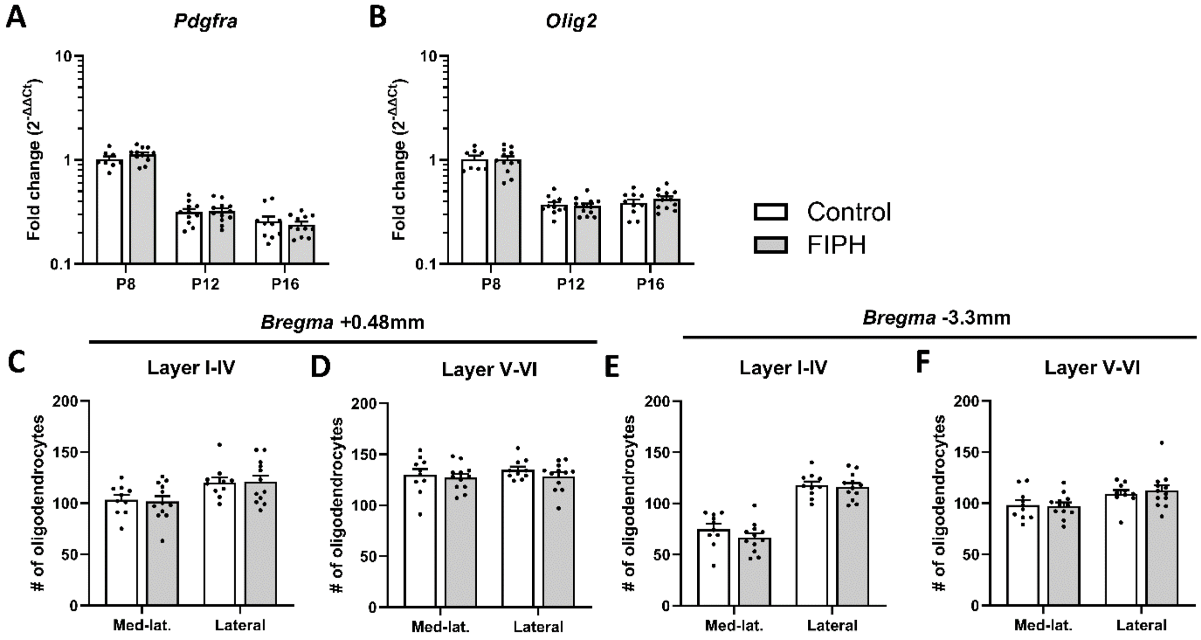

Supplementary figure 3. Total oligodendrocyte lineage cells were not different between FIPH and controls. *Markers of OPC and total lineage oligodendrocyte genes were not different between FIPH and control animals on P8, P12 or P16 at Bregma -3.3mm (A-B). The number of total oligodendrocyte lineage cells was not different between groups in any of the examined regions (C-F).*

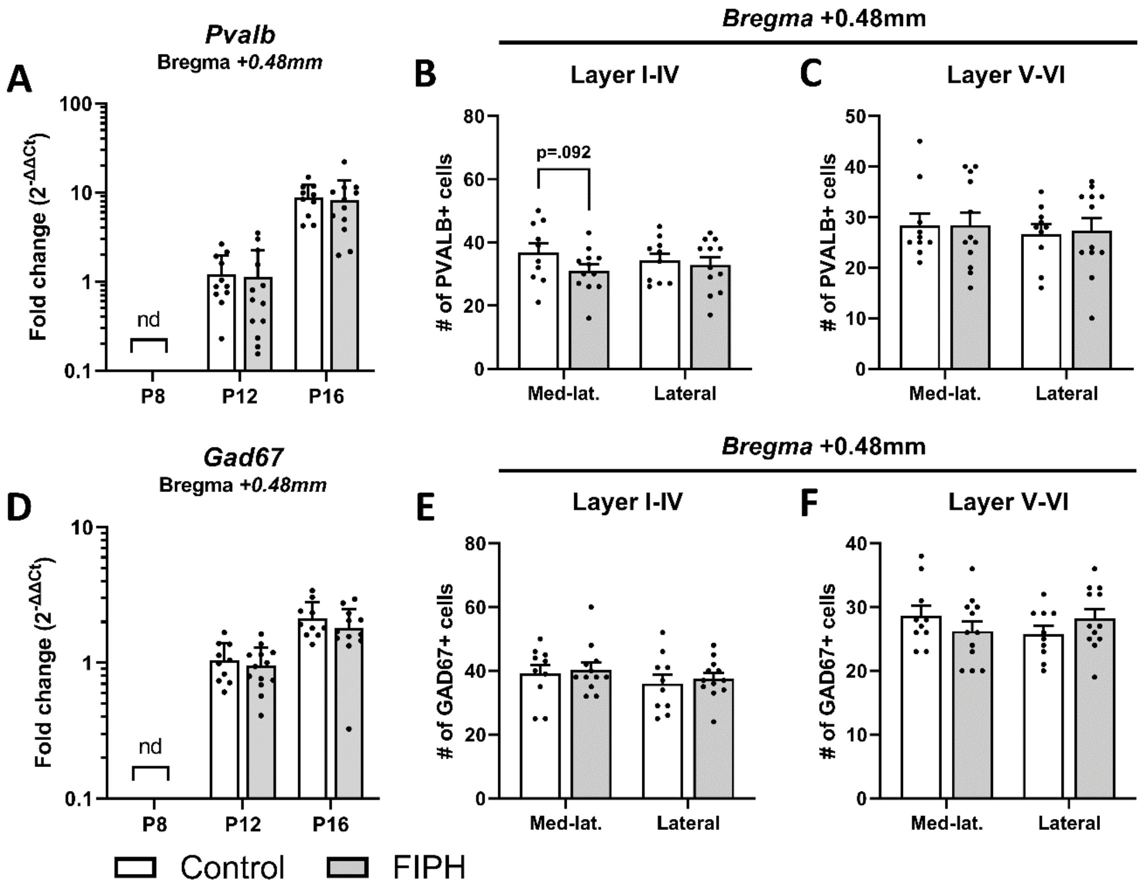

*Supplementary figure 4. **There were no differences in interneurons between FIPH and control in the examined rostral regions.** No significant differences in the Pvalb gene expression (A) or number of cells (B-C) were found at bregma +0.48mm. Similarly, no differences in Gad67 gene expression (D) or the number GAD67+ (E-F) cells between FIPH and control animals were found at bregma +0.48mm.*

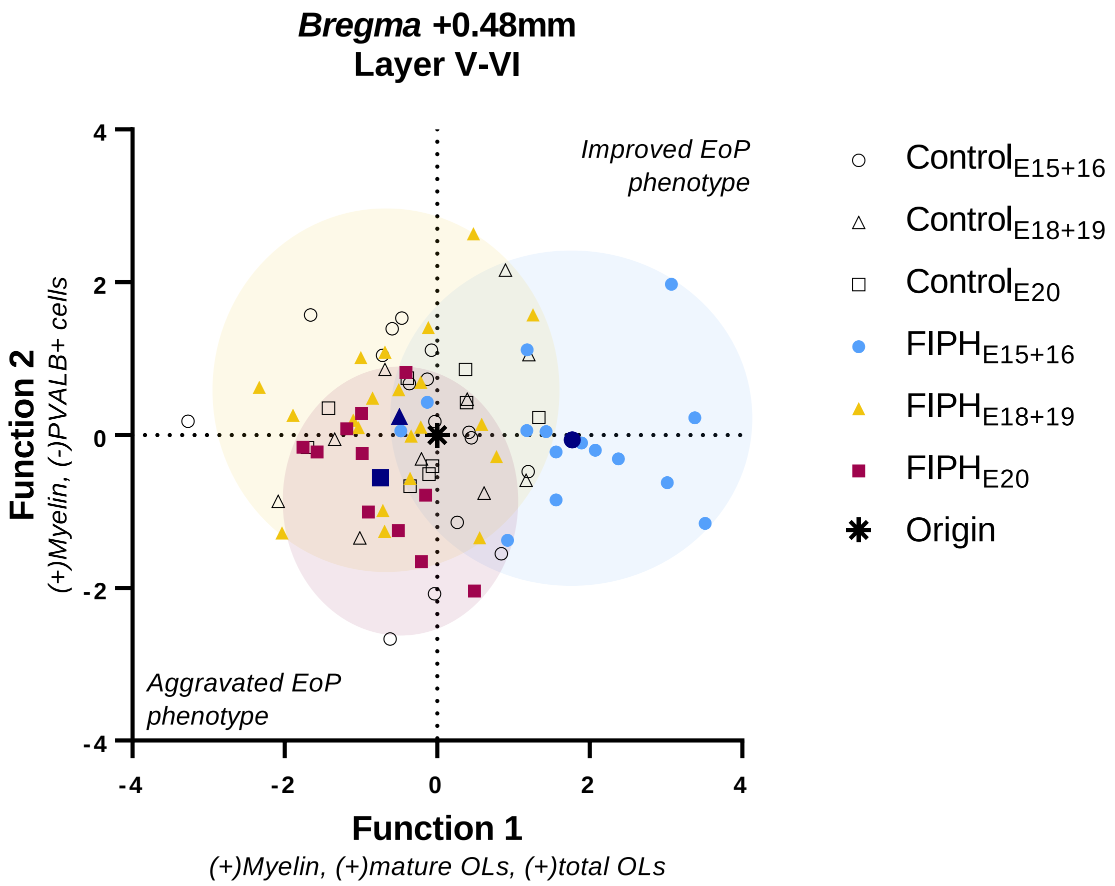

Supplementary figure 6. FIPH$_{E15+16}$ pups showed an improved EoP-phenotype in the cortex at layers V-VI. *EoP-phenotype scores in FIPH$_{E18+19}$ and FIPH$_{E20}$ were slightly reduced. ⁎ = centroid value for control animals.*

Supplementary figure 7 (next page). Inflammation at E15+16 and postnatal hypoxia causes alterations in myelin, oligodendrocytes and PVALB+ interneurons. *Global myelin-expression was decreased in layers I-IV (A, C) but not in layers V-VI (B, D) of the cortex while myelin microstructure was more dense at bregma +0.48mm (E-F) but not significantly altered at bregma -3.3mm (G-H). Conversely, the total number of oligodendrocytes (I-L) and mature oligodendrocytes (M-P) was increased, just like the number of PVALB+ interneurons in several regions (Q-T). The number of microglia branch endpoints per cell was not significantly altered between groups (U-X). Data is presented as mean ± SEM. # p < .10, * p < .05, ** p < .01, *** p < .001*

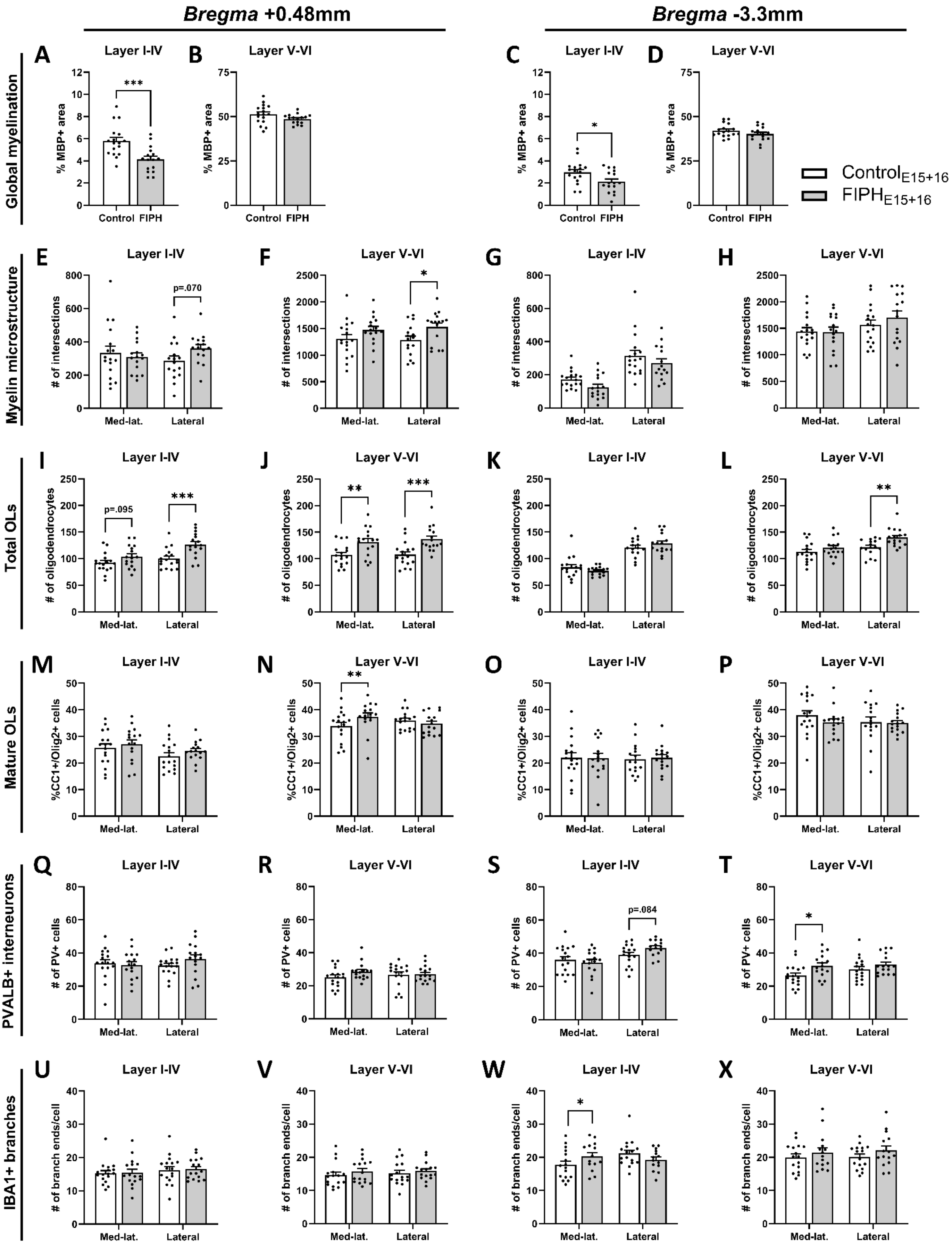

Bregma +0.48mm
Bregma -3.3mm
Global myelination
Myelin microstructure
Total OLs
Mature OLs
PVALB+ interneurons
IBA1+ branches
Layer I-IV
Layer V-VI
% MBP+ area
Control FIPH
Control E15+16
FIPH E15+16
# of intersections
Med-lat. Lateral
p=.070
p=.095
p=.084
# of oligodendrocytes
%CC1+/Olig2+ cells
# of PV+ cells
# of branch ends/cell

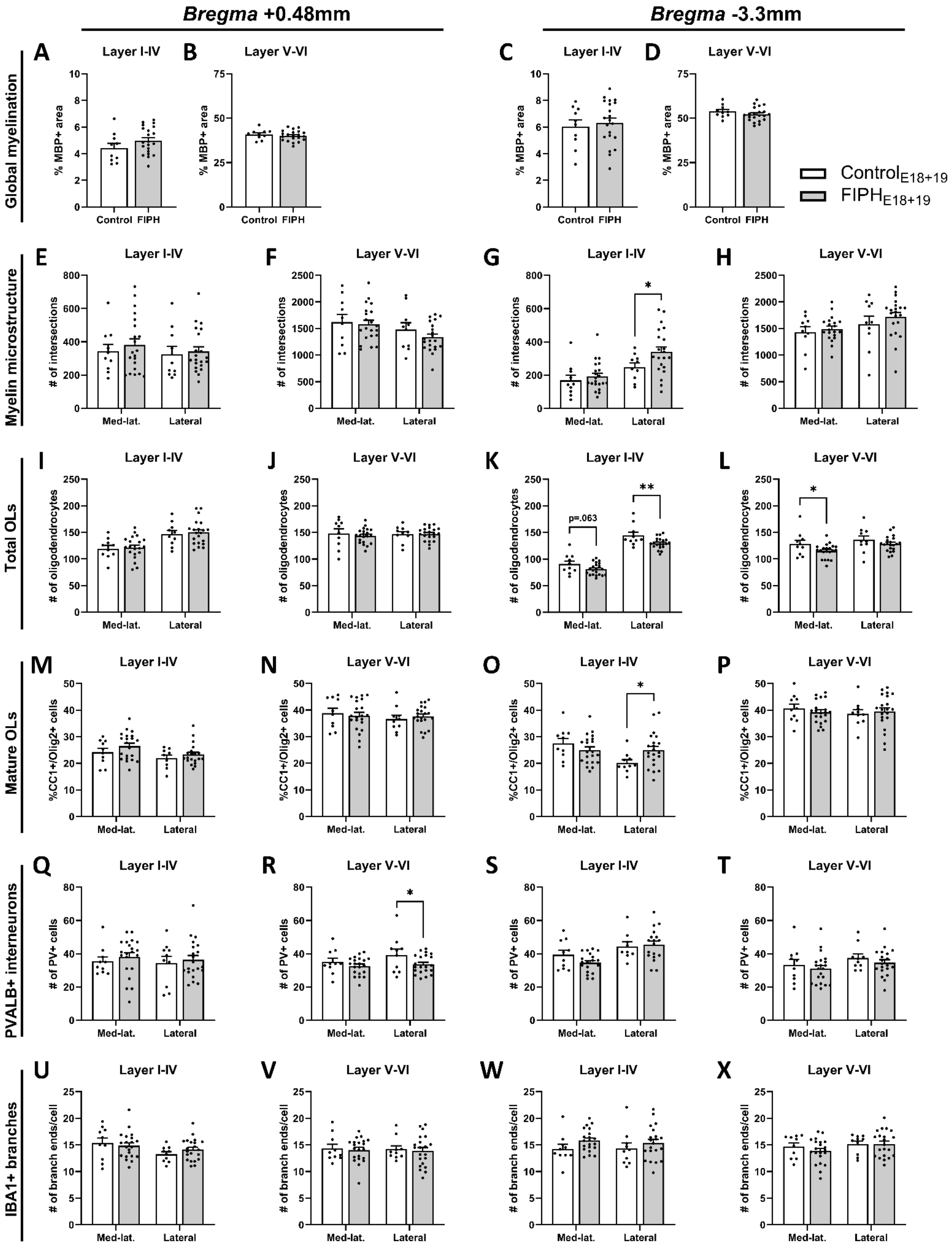
Bregma +0.48mm
Bregma -3.3mm
Global myelination
Myelin microstructure
Total OLs
Mature OLs
PVALB+ interneurons
IBA1+ branches
Layer I-IV
Layer V-VI
% MBP+ area
Control FIPH
Control$_{E18+19}$
FIPH$_{E18+19}$
# of intersections
# of oligodendrocytes
%CC1+/Olig2+ cells
# of PV+ cells
# of branch ends/cell
Med-lat.
Lateral
p=.063

Supplementary figure 8 (previous page). Inflammation at E18+19 and postnatal hypoxia causes alterations in myelin microstructure and a reduction in total oligodendrocytes and PVALB+ interneurons. *Global myelin expression was not affected in FIPH$_{E18+19}$ pups (A-D) while an increased complexity of myelin microstructure was found in the lateral cortex, layers I-IV at bregma -3.3mm (G) but not at any other location (E-F, H), corresponding to an increase in mature oligodendrocytes at this location (O) but not others (M-N, P). The total number of oligodendrocytes was reduced at bregma -3.3mm (K-L) but not at bregma +0.48mm (I-J), while reductions in the number of interneurons were found in layers V-VI at bregma +0.48mm (R) but not others (Q, S-T). Microglial morphology was not altered at P20 for FIPH$_{E18+19}$ rats compared to controls (U-X). Data is presented as mean ± SEM. # $p < .10$, * $p < .05$, ** $p < .01$, *** $p < .001$*
